# Mitochondrial dysfunction in mesenchymal stem cells impairs osteogenesis in radiation-induced bone injury via Ca^2+^-NFATc1-Fis1 pathway

**DOI:** 10.1038/s41419-025-08281-w

**Published:** 2025-12-02

**Authors:** Lin Ren, Xiaodan Chen, Ying Zheng, Jiayan Li, Jiali Yu, Linlin Ou, Gen Liu, Bin Cheng, Wei Seong Toh, Juan Xia

**Affiliations:** 1 Hospital of Stomatology, Guanghua School of Stomatology, Sun Yat-sen University, Guangzhou, China; 2 Guangdong Provincial Key Laboratory of Stomatology, Sun Yat-sen University, Guangzhou, China; 3 Department of Orthopaedic Surgery, Yong Loo Lin School of Medicine, National University of Singapore, Singapore, Singapore; 4 Faculty of Dentistry, National University of Singapore, Singapore, Singapore

**Keywords:** Mesenchymal stem cells, Cell signalling

## Abstract

Mitochondrial dysfunction of mesenchymal stem cells (MSCs) has been implicated in impaired osteogenesis, resulting in bone loss following radiation therapy. However, the underlying mechanisms remain to be fully elucidated. This study reveals the critical role of Fis1 in regulating mitochondrial dynamics and MSC osteogenesis in radiation-induced bone injury. Specifically, radiation activates Fis1 expression, which induces excessive mitochondrial fission, leading to mitochondrial fragmentation, along with reduced capacities for oxidative phosphorylation, ATP synthesis, and antioxidant defense, that collectively impairs MSC osteogenesis and results in bone loss in radiation-induced bone injury. This process involves increased calcium (Ca^2+^) influx that stimulates calcineurin (CaN) to promote nuclear factor of activated T-cells, cytoplasmic 1 (NFATc1) dephosphorylation and nuclear translocation, which in turn, activates the transcriptional expression of Fis1. Consistent with the pivotal role of Fis1 in regulating mitochondrial fission and MSC osteogenesis, inhibition of Fis1 remarkably reduced mitochondrial fragmentation, enhanced MSC osteogenesis and reduced bone loss, highlighting the therapeutic potential of targeting Fis1 in radiation-induced bone injury. Our study provides new insights into the mechanisms and therapeutic strategies for radiation-induced bone injury.

## Introduction

Radiotherapy is a common and effective treatment for various cancers [1]. However, despite its effectiveness, radiotherapy comes with a range of side effects and complications. Among all, osteoradionecrosis is a serious side effect of radiotherapy for head and neck cancers, and is considered a public health problem worldwide with a prevalence of 3-8%, adversely affecting the patients’ health recovery and quality of life [2, 3]. The condition can result in avascular necrosis of the bone tissue, leading to impaired bone healing and an increased risk of infection. Current management of osteoradionecrosis includes surgery, use of antibiotics, or hyperbaric oxygen therapy to manage and/or alleviate the symptoms. However, the treatment outcomes are not always satisfactory, underscoring the unmet need to develop new treatment strategies through improved understanding of the disease pathogenesis.

The pathological effects of radiotherapy-induced bone damage are complex and multifactorial, involving radiation-induced damage to bone tissue and surrounding vasculature [4]. As a key cellular component of bone marrow stroma, mesenchymal stem/stromal cells (MSCs) are essential for supporting hematopoiesis in the bone marrow [5] and for maintaining the postnatal bone homeostasis. Through their ability to differentiate into osteoblasts and regulate the activity of osteoclasts, MSCs play a critical role in bone formation, resorption, and repair [6]. Upon radiation, MSCs preferentially differentiate into adipocytes instead of osteoblasts, which directly impedes bone formation and indirectly aggravates bone injury through negative effects on hematopoiesis and angiopoiesis [7, 8]. Therefore, it is important to elucidate the molecular switch and mechanisms underlying the effects of radiation on MSC differentiation.

Although the deleterious effects of radiation have been largely attributed to DNA damage, reactive oxygen species (ROS) formation, and inflammation, it is increasingly recognized that mitochondria, being the central powerhouse, play an important role in mediating the cellular responses to radiation [9]. During osteogenic differentiation of MSCs, mitochondria are activated, characterized by significant upregulation of mitochondrial biosynthesis and oxidative phosphorylation (OXPHOS)-dependent metabolism [10]. Irradiated MSCs have reportedly displayed increased oxidative stress and altered electron transport chain (ETC) supercomplexes [11, 12]. However, the mechanisms underlying these changes remain to be clarified. Being highly dynamic organelles, mitochondria maintain their size, structure, and functions by undergoing coordinated cycles of fission and fusion, referred to as ‘mitochondrial dynamics.’ Mitochondrial dynamics plays an important role in maintaining the mitochondrial homeostasis and function [7]. Notably, excessive mitochondrial fission has been complicated with increased ROS damage, reduced OXPHOS and elevated apoptotic signaling [8], whereas mitochondrial elongation is associated with the increased mitochondrial biogenesis and adenosine triphosphate (ATP) production [11]. Mitochondrial fission and excessive ROS production have been observed in radiation-induced neuronal loss [12]. Therefore, we speculate that radiation alters mitochondrial dynamics and perturbs mitochondrial homeostasis, leading to MSC dysfunction and impaired osteogenesis.

In the current study, we demonstrated that radiation induced excessive mitochondrial fission through upregulating Fis1 expression via the Ca^2+^-CaN-NFATc1 pathway, which then led to mitochondrial fragmentation, impaired MSC osteogenesis and bone loss in radiation-induced bone injury. Inhibition of Fis1 remarkably reduced mitochondrial fragmentation and augmented MSC osteogenesis and bone repair in radiation-induced bone injury. Our present study highlights the therapeutic potential of targeting Fis1 in radiation-induced bone injury.

## Materials and methods

### Animal experiments

All animal studies ethically approved by Institutional Animal Care and Use Committee (IACUC), Sun Yat-Sen University (SYSU-IACUC-2022-000747, Guangzhou, China). Eight-week-old male C57BL/6 J mice (20 ± 2 g) were randomly distributed to three groups - Control, ionizing radiation (IR) and IR-siRNA (N = 6). The sample size was determined according to established protocols in prior literature [13]. Animals were randomly assigned to three experimental groups using computer-generated random numbers. To construct an animal model of radiation-induced bone injury, mice were fixed on the protective frame of a lead plate, which only bilateral tibia of the mice was fully exposed to radiation using an X-ray irradiator (Rs2000, Rad Source, USA) at a single dose of 8 Gy and distance of 40 cm. For the control and IR groups, tibia was treated with the negative control sequence (Nb. siN0000004-4-10, RiboBio, China), whereas for the IR-si*Fis1* group, tibia was treated with siRNA targeting *Fis1* (target sequence, 5’-3’: siG170718031458). 10 μL siRNA (3 nmoL/20 g body mass) was administered via intra-bone marrow injection, on the day before irradiation and once every 2 days for a total of 4 injections. In vivo animal imaging was performed using the Small Animal In Vivo Imaging System (SAIVI™, Thermo Fisher Scientific, USA) to observe the distribution of siRNA in the bone marrow cavity after siRNA with and without Cy5 fluorescent labeling was administered via tibia bone marrow injection. The animals were allowed free movement and access to standard food and water. Care and handling of the animals were performed in accordance with the Sun Yat-Sen University Animal Care and Use Committee (SYSU-IACUC-2022-000747). On day 7 after irradiation, animals were euthanized by carbon dioxide (CO_2_) inhalation and tissue samples were harvested for MSCs isolation and osteogenic ability analyses. On day 28 after irradiation, animals were euthanized by CO_2_ inhalation and tissue samples were harvested for bone mass and histological evaluation. Animals surviving the entire experimental period were collected for subsequent analysis.

### Micro-CT evaluation

The dissected tibia samples were fixed overnight with 4% paraformaldehyde and analyzed using a high resolution micro-computed tomography (micro-CT) system (SCANCO, Switzerland). Samples were scanned at a voltage of 80 kV, current of 114 μA, and resolution of 6 μm. Three-dimensional (3-D) reconstruction and quantitative analysis were performed using micro-CT packaged software. A region of interest (ROI) of 150 layers within the tibia below the growth plate was selected for measurements of the percentage of bone volume over total volume (BV/TV, %), trabecular number (Tb. N, 1/mm) and bone mineral density (BMD, mgHA/cm^3^).

### Histology, immunohistochemistry and histomorphometry

Following micro-CT evaluation, samples were decalcified for 4 weeks, before dehydrated in graded alcohols and embedded in paraffin for sectioning. Serial sections were cut at 4-µm thickness and stained with hematoxylin and eosin (H&E) for observation of general morphology. Immunohistochemical (IHC) staining was performed as previously described [14]. Briefly, the slides were incubated with antibodies for osteocalcin (OCN, 1:100, Santa Cruz, USA), tartrate-resistant acid phosphatase (TRAP, 1:100, Servicebio, China) and peroxisome proliferator-activated receptor-γ (PPAR-γ, 1:100, Santa Cruz, USA) and mitochondrial fission 1 (Fis1, 1:100, Proteintech, China) at 4 °C overnight. Thereafter, the slides were incubated with the secondary antibody at room temperature for 30 min and stained with 3,3´-diaminobenzidine (DAB) substrate, followed by nuclear counterstaining with haemoxylin. The stained slides were imaged using the Aperio AT2 scanning system (Leica, Germany). Five randomly selected fields were selected, and the mean staining density of OCN, PPAR-γ and Fis1 was measured using the ImageJ software (National Institutes of Health (NIH), Bethesda, MD, USA) and expressed as the integrated optical density (IOD) values. For quantification of TRAP, positively stained cells were counted in 5 randomly selected fields and expressed as number of TRAP^+^ cells per field.

### Cell culture and irradiation treatment in vitro

Bone marrow-derived MSCs were harvested from tibia of control (non-irradiated) and irradiated (IR) mice. Briefly, the tibia was repeatedly flushed with alpha-Minimum Essential Medium (α-MEM). The bone marrow suspension was collected and centrifuged at 1000 rpm for 5 min. After centrifugation, cell pellet was resuspended in basal growth medium comprising of α-MEM supplemented with 10% fetal bovine serum (FBS) and cultured at 37 °C in a humidified atmosphere with 5% CO_2_.

To further investigate the molecular mechanism underlying the effects of radiation on MSC osteogenesis, primary MSCs from non-irradiated mice were isolated and cultured in vitro. Cells were seeded in 6-well plates at a density of 2 × 10^5^ cells/mL. Upon 80% confluence, cells were irradiated with X-rays at a dose of 8 Gy, followed by culturing in commercially available osteogenic medium (MUXMX-04021, Cyagen, China) for another 21 days, with medium change every 3 days. After 24 h of culture, cells were harvested to assess mitochondrial dynamics, function, and associated signaling pathways. Osteogenic potential was evaluated after 14 and 21 days of culture in osteogenic medium.

### Evaluation of MSC osteogenesis

Osteogenic differentiation of MSCs was assessed by measurement of the alkaline phosphatase (ALP) activity using the 5-bromo-4-chloro-3-indolyl-phosphate/Nitro blue tetrazolium (BCIP/NBT) ALP substrate solution (Beyotime, China). Gene expression of osteogenic markers (*Runx2*, *Alp*, and *Ocn*) was measured using quantitative polymerase chain reaction (qPCR) assay. Alizarin red S (ARS) staining was performed to detect calcium deposition. Cells were fixed in 4% paraformaldehyde for 10 min and then stained with 40 mM ARS solution for 20 min at room temperature. In this assay, calcium deposits form insoluble complexes with ARS, which appear as dark red aggregates. Following staining, images were captured using a stereoscopic microscope (Leica M165 FC, Germany). For semi-quantitative analysis, ARS dye was eluted with 10% (v/v) cetylpyridinium chloride (Sigma, USA) for 5 min. The resulting solution was transferred to a 96-well plate, and optical density (OD) was measured at 562 nm using a microplate reader (BioTek Epoch 2, Agilent Technologies, USA).

### Construction of RNA interference and overexpression vectors

Specific small interfering RNAs (siRNAs) against the Fis1 and NFATc1 genes were purchased from RiboBio (Guangzhou, China). The siRNA sequences that include si-Fis1, 5’-3’, GGCTCTAAAGTATGTGCGA; and si-NFATc1, 5’-3’, GGAGGTGGAAGACGTACTT were synthesized by RiboBio. Briefly, bone marrow-derived MSCs were seeded in 6-well plates (2 × 10^5^ cells/well) and upon 80% confluence, cells were transfected with 50 nM si-Fis1 or si-NFATc1 or negative control siRNA using Lipofectamine RNAiMAX (Thermo Fisher Scientific), following the manufacturer’s instructions. To verify siRNA interference of Fis1 and NFATc1 expression, cells were harvested at 48 h post-transfection for qPCR and western blotting analyses. To investigate the effect of siRNA interference on MSCs, cells were irradiated by X-rays at a dose of 8 Gy after transfection. Cells were then cultured with osteogenic medium for another 21 days

The mouse NFATc1 (NCBI ID: 18018)-overexpressing vector (plasmid) was constructed and packaged into lentiviral particles by Suzhou GeneChem Co. MSCs were seeded in 6-well plates at a density of 2 × 10⁵ cells per well. Upon reaching 90% confluence, the original lentiviral stock was diluted 10-fold in culture medium, and 100 μL of the diluted viral supernatant was add to each well. Control wells received virus-free medium. After 24 h of incubation, the medium was replaced with fresh complete growth medium. Transduced cells were then cultured for an additional 48 h before proceeding with experimental analyses. Un-transduced cells served as blank controls throughout the experiment.

### ROS and SOD measurements

A diacetyldichlorofluorescein (DCFH-DA) fluorescent redox probe was used to detect the reactive oxygen species (ROS) in cells. Briefly, the cells were incubated with α-MEM containing 10 μM DCFH-DA probe (Beyotime) at 37 °C for 20 min. The fluorescence intensity of cells was then analyzed by flow cytometry (Beckman, USA) or visualized using confocal laser scanning microscope (CLSM, Olympus FV3000, Japan). In addition, cells were collected for measurement of the superoxide dismutase (SOD) activity using the total SOD assay kit (Beyotime), following the manufacturer’s instructions.

### Mitochondrial respiration test

The oxygen consumption rate (OCR) of MSCs was monitored in real-time using a XF96 Extracellular Flux Analyzer (Agilent, USA) with the Seahorse XF Cell Mito Stress Test Kit (Agilent), following the manufacturer’s instructions. Briefly, MSCs were seeded in the XF-96 microplate at a concentration of 1 × 10^4^ cells/well. Prior to the assay, cells were washed, and medium was replaced with the Seahorse XF DMEM (103575-100, Agilent Technologies, USA) and incubated in a non-CO_2_ incubator at 37 °C for 1 h. Bioenergetic profiles were measured by serial injections of oligomycin (1.5 μM), fluorocarbonyl phenylhydrazone (0.5 μM) and rotenone/antimycin A (0.5 μM). Readings were recorded for approximately 80 min.

For ATP measurement, cells were lysed and samples were analyzed using the ATP assay kit (Beyotime). The relative light unit (RLU) values of samples were measured using a luminometer (Bio-Tek, USA) and converted to specific ATP concentrations using a standard provided in the kit. To evaluate the mitochondrial membrane potential (MMP), cells were incubated with 50 nM tetramethylrhodamine ethyl ester perchlorate (TMRE, Beyotime) for 30 min. Images were then taken using the CLSM (Olympus FV3000, Japan).

### Morphological analysis of mitochondria

MSCs were fixed for 1 h with 2.5% glutaraldehyde, before suspended in fresh fixation buffer for 10 min. Cells were then fixed with 1% osmic acid for 3 h, dehydrated through a series of graded ethanol (50%, 70%, 90%), and then transferred to 100% acetone for 20 min. Next, cells were embedded with pure acetone and embedding solution. Samples were sectioned at 50–60 nm thickness using Ultracut (Leica, Wetzlar, Germany). Sections were then double-stained with 3% uranyl acetate-lead citrate for observation under high resolution transmission electron microscopy (TEM) using Tecnai G2 Spirit TWIN microscope (FEI, USA). Measurements of minimum diameters (c-Min) and maximum (c-Max) of mitochondria were performed using the TEM images, and the ratio of c-Min/c-Max was calculated to evaluate the globularity. Mitochondria with spherical or punctate morphology exhibited aspect ratios close to 1, while elongated or rod-shaped mitochondria displayed lower aspect ratios. In separate experiments, MSCs were incubated with 100 nM MitoTracker™ Red (Thermo Fisher Scientific) for 30 min to stain for active mitochondria. Nuclear staining was performed using the Hoechst solution (Beyotime). Images were taken using a CLSM (Olympus FV3000). The mitochondrial morphology was analyzed using the ImageJ-MiNA (Mitochondrial Network Analysis) toolset (NIH) [15]. Briefly, ImageJ-MiNA classified mitochondria structures into Networks (interconnected tubules with ≥3 branches) and Individuals (isolated, unbranched puncta), and calculated key parameters including: Number of Individuals/Networks, Mean Network Size and Mean Branch Length. For our analysis, we used the proportion of Individuals (Individuals/total) and Mean Network Size as primary metrics to characterize the mitochondrial morphology.

### RNA sequencing

RNA was isolated using TRIzol reagent (Thermo Fisher Scientific). Samples were stored in liquid nitrogen and transported to BGI Genomics, Shenzhen, China. RNA sequencing libraries were constructed and deep sequencing was performed using the BGISEQ-500RNA seg platform. Sequencing data were filtered with SOAPnuke, and a heatmap was drawn using Pheatmap according to the gene expression of different samples. Differential expression analysis was performed using DESeq2 with a Q value ≤ 0.05. Quantitative analysis of genes, including Kyoto Encyclopedia of Genes and Genomes (KEGG) analyses, cluster analysis, and signaling pathway map illustration, was performed on the BGI multi-omics system. KEGG enrichment analyses of differentially expressed genes (DEGs) were performed using Phyper based on hypergeometric test. Significant levels were corrected by Q value with a rigorous threshold (Q value ≤ 0.05) by Bonferroni correction.

### Ca^2+^ and calcineurin (CaN) detection

Intracellular calcium (Ca^2+^) concentration was analyzed by flow cytometry. Briefly, cells were treated with 2 μM Fluo-4 AM fluorescent probe (Beyotime) in culture medium for 30 min. After washing with phosphate-buffered saline (PBS), the fluorescence intensity of cells was analyzed at 494 nm using ‌CytoFLEX flow cytometry (Beckman, USA). As per the manufacturer’s instructions, the calcineurin (CaN) activity was assayed using the CaN assay kit (Nanjing Jiancheng, China) and OD readings were taken at 636 nm using a microplate reader (BioTek Epoch 2).

### Chromatin immunoprecipitation (ChIP) assay

ChIP was conducted using a ChIP Assay Kit (P2078, Beyotime, China) according to the manufacturer’s instructions. Briefly, cells were cross-linked with a 37% formaldehyde solution for 10 min at room temperature and quenched with 125 mM glycine. Ultrasonication was used to obtain DNA fragments within 1000 bp. Samples were subjected to immunoprecipitation using anti-NFATc1 antibody (Santa Cruz) and IgG (Proteintech, USA). Protein A + G Agarose/Salmon Sperm DNA (Beyotime) was used to precipitate proteins or corresponding complexes recognized by the primary antibody. DNA complexes obtained by ChIP were further purified using a DNA purification kit (Beyotime). The NFATc1-binding site in the *Fis1* promoter (-1868 bp to -1877 bp) with the sequence of GAAAGGAAAA was predicted using the JASPAR database. The *Fis1* primers were designed and synthesized by Servicebio Technology. The immuno-precipitated DNAs were analyzed using qPCR.

### RNA extraction and qPCR

Total RNA was isolated from cells using an RNA Quick Purification Kit (RN001, ESscience, China), and reverse transcribed to cDNA using Hifair® III reverse transcriptase (Yeasen Biotech). qPCR was performed with Hieff® qPCR SYBR Green Master Mix using the QuantStudio 5 Flex System (Applied Biosystems, USA). The qPCR cycling condition comprised an initial denaturation at 95 °C for 5 min followed by 40 cycles of 95 °C for 10 s, 60 °C for 20 s, and 72 °C for 20 s. Relative mRNA expression levels were normalized to *β-actin*, calculated using the 2^−∆∆Ct^ method and finally expressed as fold changes. Primer sequences are listed in Table S1.

### Western blotting

Cells were lysed using radioimmunoprecipitation assay (RIPA) buffer supplemented with protease and phosphatase inhibitors. To assess the levels of NFATc1 in nucleus and cytoplasm, nuclear and cytoplasmic proteins were isolated using the Nuclear and Cytoplasmic Protein Extraction Kit (Beyotime). Protein extracts (40 µg) were separated using 4-20% gel electrophoresis (FuturePAGE™, ACE Biotechnology, China) and then transferred to polyvinylidene difluoride (PVDF) membranes. Subsequently, membranes were blocked with 5% skim milk, followed by incubation with primary antibodies for Fis1 (1:1000, Proteintech, USA), phospho-dynamin-related protein 1 (p-DRP1(Ser616), 1:1000, Cell Signaling Technology (CST), USA), NFATc1 (1:1000, Affinity, China), β-actin (1:5000, Emarbio, China) and histone 3 (1:5000, CST, USA) at 4 °C overnight. The membranes were then incubated with horseradish peroxidase (HRP)-conjugated secondary antibodies. Visualization of the protein bands was performed by incubating with the chemiluminescence substrate provided in an enhanced chemiluminescence (ECL) kit (Absin, China) and captured using an enhanced ImageQuant LAS4000 chemiluminescence detection system (GE Healthcare, USA).

### Statistical analysis

Blinding was implemented during outcome assessment. Investigators performing data analysis were unaware of group allocations. All data are presented as mean ± standard deviation (SD). For in vitro experiments, the results were based on triplicate analysis from at least three independent experiments. Statistical analyses were performed using SPSS 20.0 (IBM, USA). Data normality was assessed with the Shapiro-Wilk tests. For comparisons of two groups, unpaired Student’s *t*-test was used. For comparisons for multiple groups, one-way ANOVA followed by Tukey’s multiple comparison test was used. *P*-value of < 0.05 was considered to be statistically significant.

## Results

### Radiation disrupts bone homeostasis and inhibits MSC osteogenesis

**Fig. 1:**
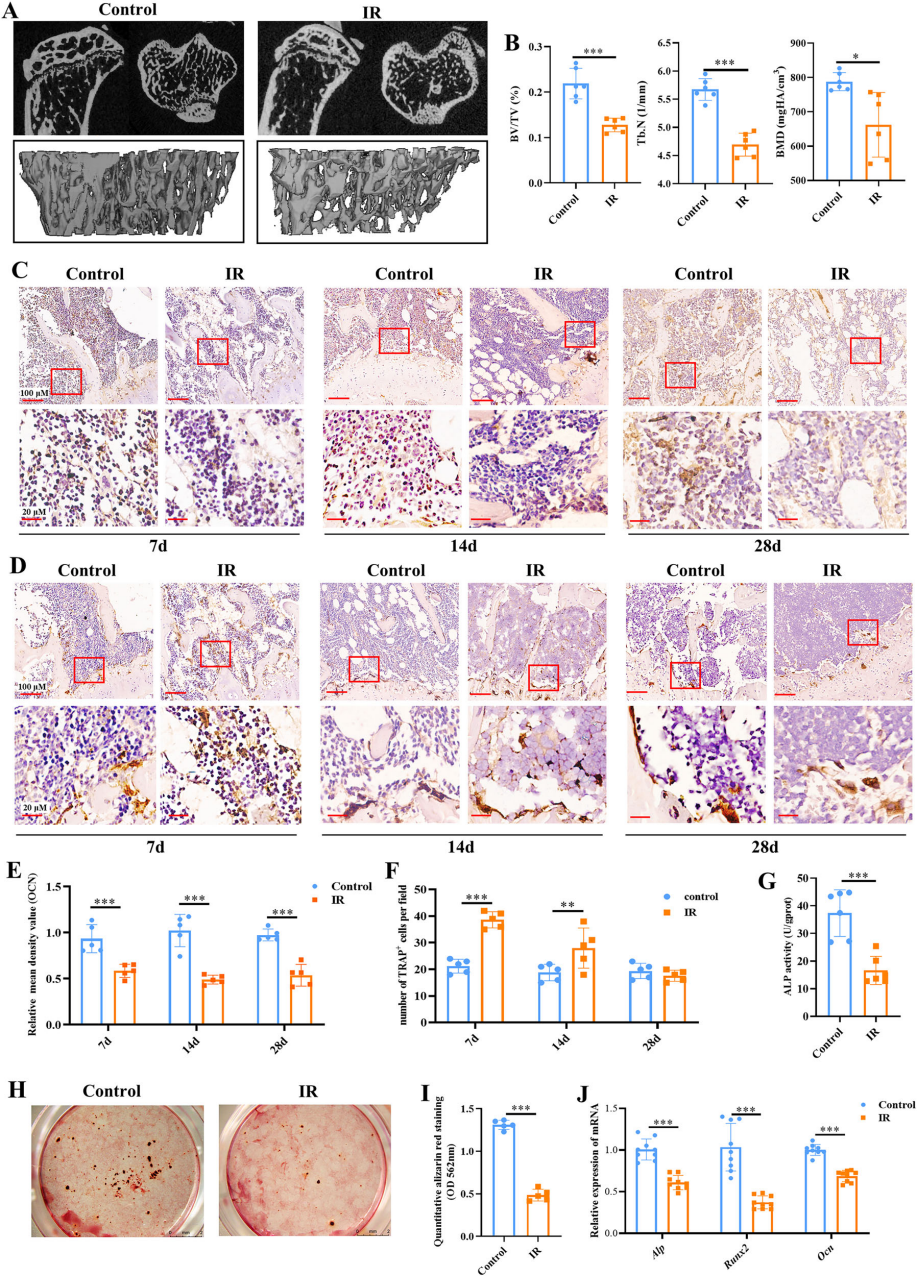
Radiation induced bone loss and impaired MSC osteogenesis. Eight-week-old male C57BL/6J mice were randomly distributed to two groups – Control and ionizing radiation (IR) group. IR mice were irradiated at a single dose of 8 Gy. **A** Representative micro-CT images of tibia showing the sagittal and sagittal and the region of interest (ROI, bottom panel). **B** Quantitative analysis of BV/TV, Tb.N and BMD. Representative images of OCN (**C**) and TRAP (**D**) staining of the tibial samples. Scale bars, 100 µm or 20 µm. Immunohistochemical staining and semi-quantitative analysis of OCN stained areas (**E**) and TRAP^+^ cells (**F**). Bone marrow-derived MSCs were harvested from tibia of control and IR mice and evaluated for their osteogenic differentiation capacity, including the measurement of ALP activity (**G**) Alizarin red S (ARS) staining (**H**) and semi-quantification of eluted dye (**I**) and qPCR analysis of mRNA levels of *Alp, Runx2* and *Ocn* (**J**). Data are presented as mean ± SD, *p < 0.05, **p < 0.01, ***p < 0.001. Statistical analyses were determined by unpaired Student’s *t* test.

On day 28 following radiation, animals were euthanized and tibial samples were harvested for micro-CT evaluation of the bone mass, structure and mineralization. Relative to the control mice, the irradiated (IR) mice had a dramatic bone loss, as evidenced by a significant decrease in BV/TV (*p* < 0.001), Tb.N. (*p* < 0.001) and BMD (*p* < 0.05) (Fig. 1A, B). Consistent with the micro-CT results, histological evaluation by H&E staining revealed thinning of bone struts within the trabecular bone, and increase in bone porosity and bone marrow adiposity in the IR group, as compared to the control group (Fig. S1). Further immunohistochemical staining for OCN (a marker for osteoblasts) and TRAP (a marker for osteoclasts) revealed detrimental effects of irradiation on bone formation and remodeling (Fig. 1C, D). Notably, we observed a significant decrease in IOD for OCN as early as day 7 that persisted to day 28 in the IR mice, as compared to the control mice (*p* < 0.001) (Fig. 1E). In contrast, the number of TRAP^+^ cells increased at day 7 (*p* < 0.001) and persisted to day 14 (*p* < 0.01) in the IR group, but declined to level similar to the control group at day 28 (Fig. 1F). These results indicated that radiation induced bone damage by impairing osteogenic new bone formation and elevating osteoclastic bone resorption, and impaired osteogenesis appeared to be a more critical factor for persistent bone loss following irradiation.

We then isolated primary bone marrow-derived MSCs from both control and irradiated mice. Flow cytometry analysis showed comparable CD44⁺ expression: 88.4% in control vs. 85.9% in IR mice (Fig. S2), maintaining the expression of the surface marker, typical of MSCs [16]. No significant difference was observed, indicating that radiation exposure did not alter CD44 expression. This finding is consistent with a previous report showing that MSCs retain their surface markers after particle radiation [17]. Consistent with the impaired bone formation observed in vivo, we observed that MSCs isolated from IR mice had lower ALP activity, reduced calcium deposition, and downregulated expression of osteogenic genes including *Alp*, *Runx2*, and *Ocn*, as compared to MSCs from the control mice (*p* < 0.001) (Fig. 1G-J). These results suggest that radiation impairs new bone formation by impeding MSC osteogenesis.

### Radiation induces mitochondrial dysfunction in MSCs

**Fig. 2:**
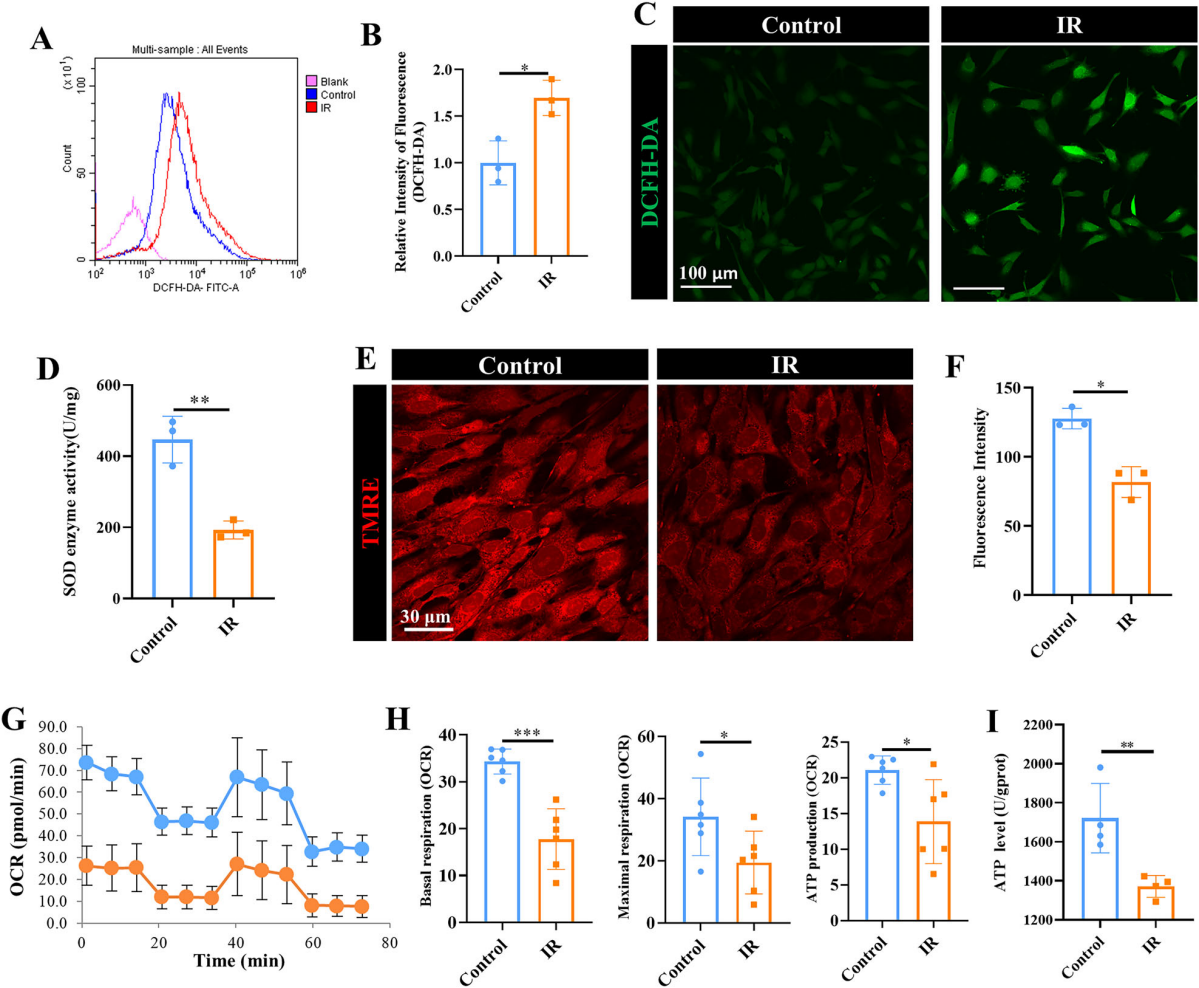
Radiation induced oxidative stress damage and OXPHOS suppression in mitochondria. Bone marrow-derived MSCs were harvested from tibia of control and IR mice and evaluated for their mitochondrial function. Analysis of ROS production by DCFH-DA staining using flow cytometry (**A**, **B**) and representative images (**C**). Scale bars, 100 µm. **D** Quantification of SOD enzyme activity level. TMRE staining (**E**) and semi-quantitative analysis of fluorescence intensity for mitochondrial membrane potential (MMP) assessment (**F**). Representative images. Scale bars, 30 µm. Metabolic analysis using Seahorse analyzer showing OCR curve (**G**) and measurement of basal respiration, maximal respiration and ATP-linked respiration (**H**). **I** Quantitative measurement of ATP level. Mean ± SD, *p < 0.05, **p < 0.01, ***p < 0.001. Statistical analyses were determined by unpaired Student’s *t* test.

Next, we investigated the mechanisms underlying impaired MSC osteogenesis in radiation-induced bone injury. We first performed DCFH-DA fluorescent assay and SOD assay to measure the ROS levels and SOD activities, respectively. Notably, MSCs from IR mice had higher ROS level over that of MSCs from control mice (*p* < 0.05) (Fig. 2A-C). On the contrary, SOD activity was significantly lower in MSCs from IR mice, as compared to MSCs from control mice (*p* < 0.01) (Fig. 2D). Analysis of the mitochondria membrane potential (MMP) by TMRE staining revealed significantly reduced MMP in MSCs from IR mice, as compared to their control counterpart, suggesting reduced mitochondrial respiration following radiation (*p* < 0.05) (Fig. 2E, F). Metabolic analysis using Seahorse analyzer further showed reduced oxygen consumption rate (OCR) of basal (*p* < 0.001), maximal respiration (*p* < 0.05) and ATP production (*p* < 0.05), indicating impaired oxidative phosphorylation (OXPHOS) in MSCs from IR mice, as compared to their counterpart control (Fig. 2G, H). Additionally, ATP assay confirmed the reduced level of ATP synthesis in MSCs from IR mice, as compared to those from control mice (*p* < 0.01) (Fig. 2I). These findings provide compelling evidence that radiation resulted in excessive ROS production, MMP loss and ATP deficit, leading to mitochondrial dysfunction of MSCs.

### Radiation promotes mitochondrial fission and upregulates Fis1 expression

**Fig. 3:**
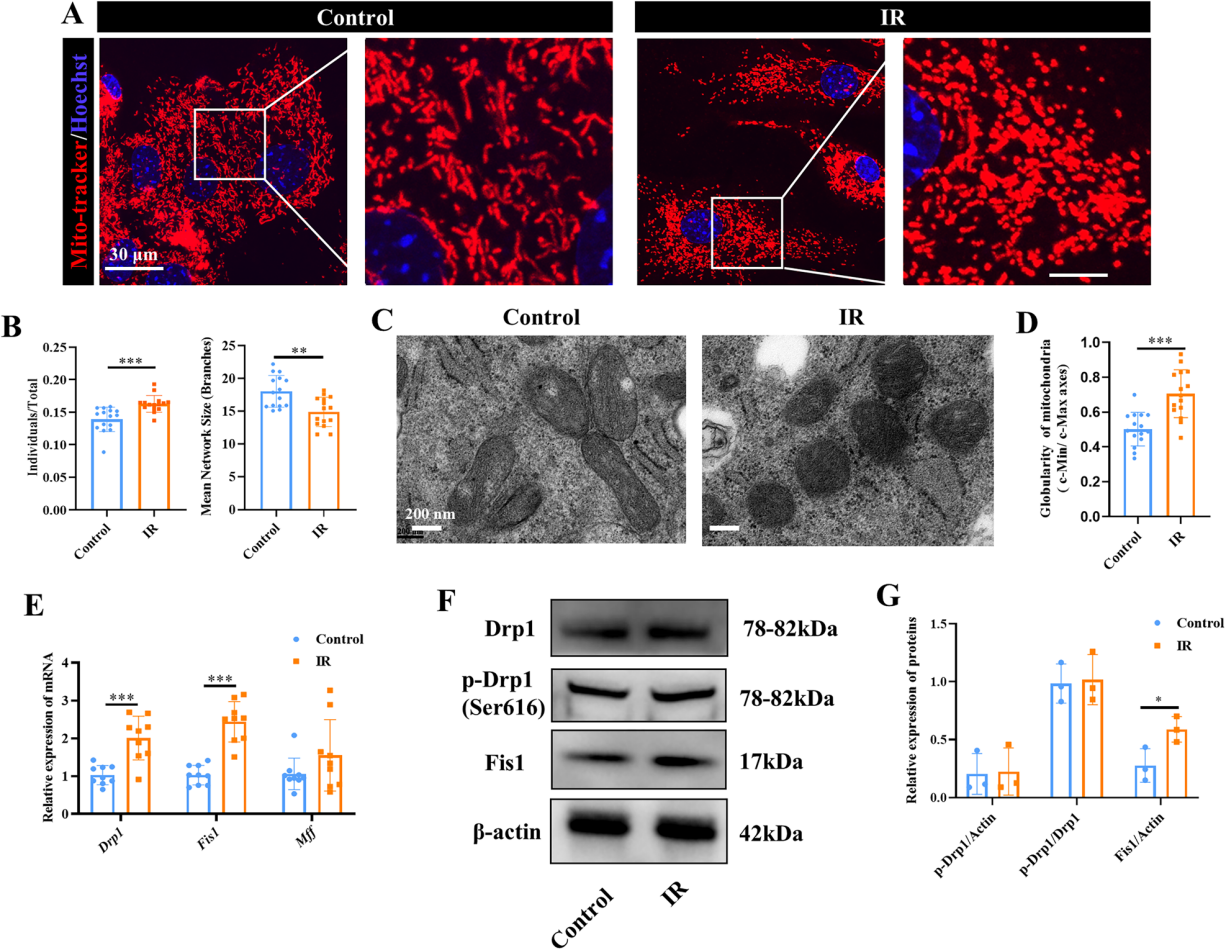
Radiation increased mitochondrial fission and Fis1 expression. Bone marrow-derived MSCs were harvested from tibia of control and IR mice and evaluated for their mitochondrial dynamics. **A** MitoTracker™ Red staining for mitochondria in MSCs. Representative images. Scale bars, 30 µm. **B** Semi-quantitative analysis of mitochondrial individuals (left) and mitochondrial network (right) using ImageJ-MINA. **C** TEM analysis of mitochondrial morphology. Representative images. Scale bars, 200 nm. **D** Globularity analysis of the mitochondria based on TEM images. **E** qPCR analysis of *Drp1*, *Fis1* and *Mff*. Western blotting (**F**) and semi-quantitative analysis (**G**) of p-Drp1 and Fis1. Mean ± SD, *p < 0.05, **p < 0.01, ***p < 0.001. Statistical analyses were determined by unpaired Student’s *t* test.

Mitochondrial dynamics, which involves the processes of fission, fusion, biogenesis and mitophagy, plays a critical role in maintaining mitochondrial homeostasis and function [18]. This prompted us to determine if mitochondrial dynamics was altered by radiation. We performed mitochondrial staining using MitoTracker™ Red followed by analysis of mitochondrial morphology using ImageJ-MiNA. Radiation exposure markedly increased mitochondria fission, as evidenced by significantly a higher number of mitochondrial individuals (*p* < 0.001) and a smaller mean network size (*p* < 0.01) in MSCs from IR mice, as compared to those from control mice (Fig. 3A, B). Additionally, TEM analysis revealed that mitochondria in MSCs from IR mice appeared spherical with obscured cristae structures, whereas mitochondria in MSCs from control mice appeared tubular with distinct cristae structures (Fig. 3C). Quantitative analysis of TEM images further supported these observations, showing a significant increase in the aspect ratio (c-Min/c-Max) and globularity of mitochondria in MSCs from IR mice, consistent with the increased mitochondrial fission and a shift toward a more spherical morphology (*p* < 0.001) (Fig. 3D). Consistent with the structural mitochondrial alterations observed in MSCs from IR mice, genes associated with mitochondrial fission, including *Drp1* and *Fis1*, were significantly upregulated at mRNA level (*p* < 0.001) (Fig. 3E). At protein level, Fis1 but not p-Drp1(Ser616) was significantly upregulated in MSCs from IR mice, as compared to those from control mice (*p* < 0.05) (Fig. 3F, G).

To ascertain the effects of radiation on MSCs, an MSC model of radiation damage was established in vitro. We first established a dose gradient of 0, 4, 8 and 16 Gy to identify the radiation dose that caused injury to MSCs in vitro. Alizarin Red S quantification revealed a clear dose-dependent impairment of osteogenic potential. An 8 Gy radiation dose was found to induce a significant impairment of osteogenic potential (65% reduction vs control, *p* < 0.001) (Fig. S3). These findings aligned with our observations that 8 Gy radiation was sufficient to consistently induced bone loss in our murine models in vivo. Therefore, 8 Gy was selected for subsequent experiments. Consistent with the above observations in MSCs isolated from the IR mice, MSCs displayed increased mitochondrial fission and upregulation of Fis1 (*p* < 0.01) (Fig S4A–E) following irradiation in vitro. Relative to non-irradiated MSCs, irradiated MSCs also showed elevated ROS level (*p* < 0.001) (Fig. S5A), decreased SOD activity (*p* < 0.001) (Fig. S5B), reduced OCR of basal, maximal respiration, and ATP production (*p* < 0.001), as well as ATP level (*p* < 0.001) (Fig. S5C–E), indicating suppressed OXPHOS in irradiated MSCs. Together, these findings suggest that mitochondrial dysfunction of MSCs following radiation is likely due to alteration in mitochondrial dynamics with increased mitochondrial fission.

### Fis1 inhibition alleviates radiation-induced mitochondrial dysfunction and impaired MSC osteogenesis

**Fig. 4:**
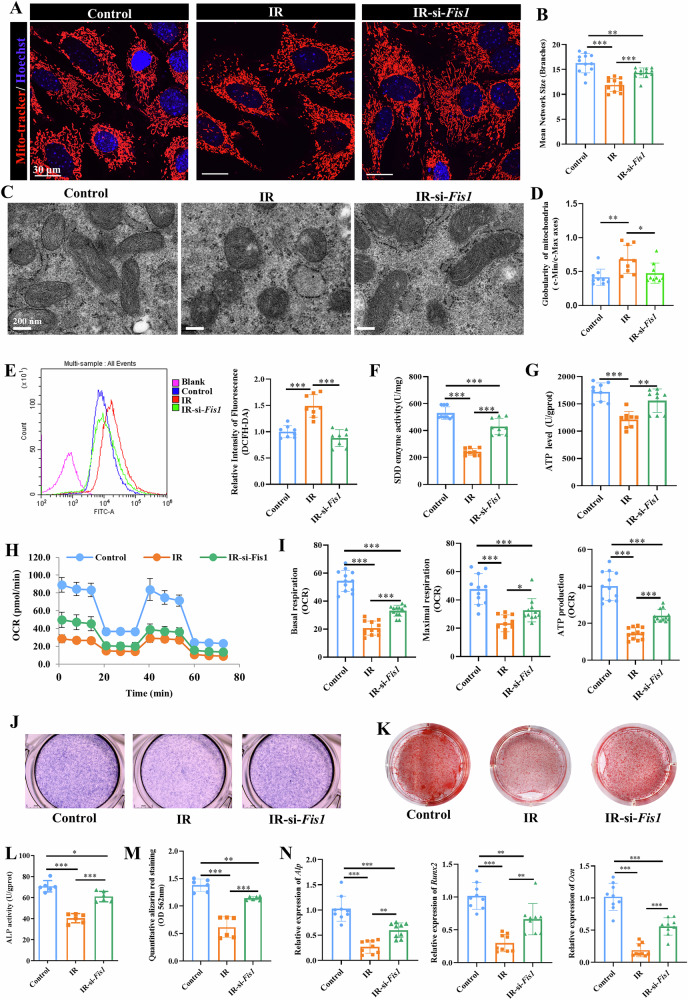
Inhibition of Fis1-mediated mitochondrial fission restored mitochondrial homeostasis and improved osteogenic differentiation capacity of irradiated MSCs. An MSC model of radiation damage was established in vitro. Fis1 was inhibited using siRNA (si-*Fis1*) to investigate whether Fis1-regulated mitochondrial fission influences MSC osteogenesis following radiation. **A** MitoTracker™ Red staining of mitochondria in MSCs. Representative images. Scale bars, 30 µm. **B** Semi-quantitative analysis of mitochondrial network using ImageJ-MINA. **C** TEM analysis of mitochondrial morphology. Representative images. Scale bars, 200 nm. **D** Globularity analysis of the mitochondria based on TEM images. **E** Analysis of ROS production by DCFH-DA fluorescent assay using flow cytometry. **F** Measurement of SOD activity. **G** Quantitative measurement of ATP level. Metabolic analysis using Seahorse analyzer showing OCR curve (**H**) and quantification of basal respiration, maximal respiration and ATP-linked respiration (**I**). ALP staining (**J**) and quantitative analysis of ALP activity (**L**). ARS staining (**K**) and semi-quantification of eluted dye (**M**). **N** qPCR analysis of *Alp, Runx2, and Ocn*. Mean ± SD, *p < 0.05, **p < 0.01, ***p < 0.001. Statistical analyses were determined by one-way ANOVA followed by Tukey’s multiple comparison test.

Although suppressed OXPHOS following radiation has been known to impede MSC osteogenesis, the effects of altered mitochondrial dynamics on MSC osteogenesis remain to be demonstrated. We therefore tested if mitochondrial fission regulated by mitochondrial fission 1 (Fis1) could influence MSC osteogenesis following radiation. By means of siRNA inhibition of Fis1 (Fig. S6A, B**)**, we showed that Fis1 inhibition alleviated mitochondrial fission of irradiated MSCs, as evidenced by increase in the network size by MitoRed staining (Fig. 4A, B). Consistent with this, TEM image analysis confirmed that Fis1 inhibition reduced the mitochondrial aspect ratio (c-Min/c-Max) and degree of globularity in irradiated MSCs (*p* < 0.05) (Fig. 4C, D), indicating reduced mitochondrial fission. Additionally, Fis1 inhibition was able to reduce the ROS level, partially restore the SOD activity, and increase the ATP synthesis in irradiated MSCs, attaining ROS and ATP levels close to that of non-irradiated MSCs (*p* < 0.01) (Fig. 4E–G). Further analyses also revealed significant improvements in OCR of basal and maximal respiration, and ATP production in irradiated MSCs with Fis1 inhibition (*p* < 0.05) (Fig. 4H, I).

Upon osteogenic differentiation, irradiated MSCs with Fis1 inhibition showed significantly higher levels of ALP activity, calcium deposition (*p* < 0.05) (Fig. 4J-M**)** and collagen I expression (Fig. S7) as compared to irradiated MSCs. These findings were further supported by our gene expression results showing significantly higher expression levels of osteogenic gene markers, including *Alp, Runx2, and Ocn* in irradiated MSCs with Fis1 inhibition (*p* < 0.01) (Fig. 4N). These findings suggest that Fis1 inhibition alleviated radiation-induced mitochondrial dysfunction and impaired osteogenesis by reducing excessive mitochondrial fission.

### Radiation upregulates Fis1 expression via activation of the Ca^2+^-CaN-NFATc1 pathway

**Fig. 5:**
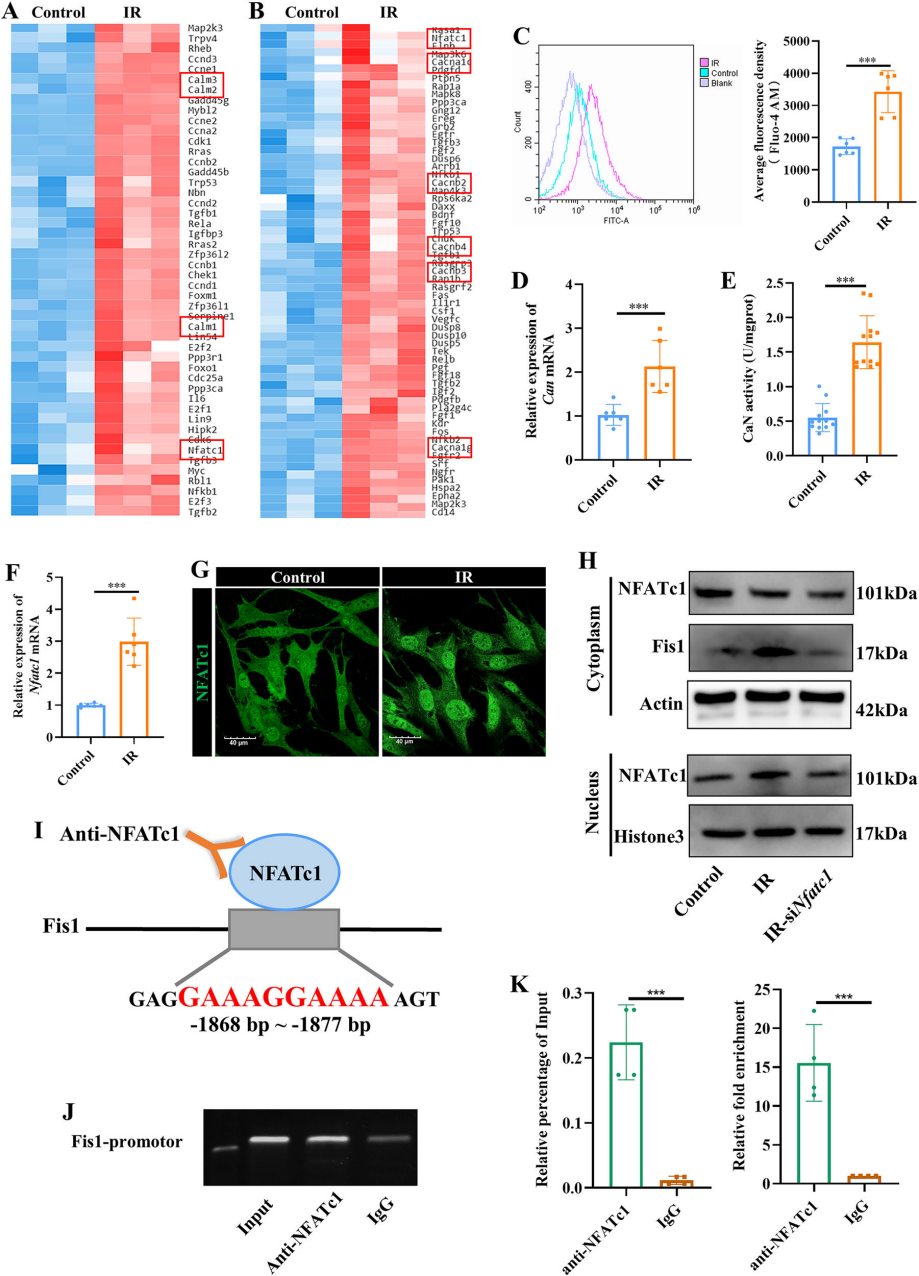
Radiation promoted expression of Fis1 by activating the Ca^2+^-CaN-NFATc1 signaling pathway in MSCs. An MSC model of radiation damage was established in vitro. RNA sequencing data from irradiated (IR) and control MSCs. Cluster heatmap of significantly different genes related to the cellular senescence pathway (**A**) and MAPK pathway (**B**). **C** Level of cytoplasmic Ca^2+^ detected by flow cytometry. **D** qPCR analysis of mRNA level of *Can*. **E** Quantification of CaN enzyme activity. **F** qPCR analysis of *Nfatc1*. **G** Immunofluorescence staining of NFATc1. Representative images. Scale bars, 40 µm. **H** Western blotting analysis showing NFATc1 and Fis1 expression in the nucleus or cytoplasm following siRNA inhibition of *Nfatc1*. **I** Predicted NFATc1-binding site is located at positions -1868 bp ~ -1877 bp of the *Fis1* promoter, with the sequence of GAAAGGAAAA. ChIP analysis showing NFATc1 protein binding to *Fis1* promoter. NFATc1-bound DNA was detected by agarose gel electrophoresis (**J**). qPCR analysis of NFATc1-bound DNA showed the enrichment abundance between different groups (**K**). Mean ± SD, ***p < 0.001. Statistical analyses were determined by unpaired Student’s *t* test.

To delve into the mechanisms underlying Fis1 upregulation, RNA sequencing was performed (Fig. S8A). Of 4892 differentially expressed genes (DEGs) between the irradiated and non-irradiated MSCs, 2484 genes were found upregulated in the irradiated cells (Fig. S8B). KEGG enrichment analysis identified top 20 signaling pathways with significant enrichment in the irradiated MSCs (Fig. S8C). Focusing on cellular senescence and mitogen-activated protein kinase (MAPK) pathways as the major pathways regulating mitochondrial dynamics [19, 20], further cluster analysis identified DEGs such as calmodulin gene (*Calm1/2/3*) and calcium channel gene α/β (*Cacna1c/1* *g*, *Cacnb2/3/4*) involved in Ca^2+^-related signaling that were significantly upregulated in irradiated cells (Fig. 5A, B). Concurrently, nuclear factor of activated T-cells, cytoplasmic 1 (*Nfatc1*) was also found elevated in both cell senescence and MAPK pathways (Fig. 5A, B). These findings suggest the involvement of NFATc1 activation upon radiation through a calcium-dependent signaling pathway.

Thus, the level of intracellular Ca^2+^ was measured. Irradiated MSCs showed increased level of Ca^2+^ as compared to the non-irradiated cells, indicating increased Ca^2+^ influx in MSCs upon radiation (*p* < 0.001) (Fig. 5C). Consistent with the role of Ca^2+^ influx in CaN activation, irradiated MSCs exhibited higher mRNA expression of *Can* (*p* < 0.001) (Fig. 5D) and higher CaN enzyme activity (*p* < 0.001) (Fig. 5E) as compared to the non-irradiated cells. These findings were supported by further KEGG pathway analysis that showed significant upregulation of *Can* and genes related to the calcium channels (Fig. S8D). Next, we verified that the increased Ca^2+^ influx and CaN activity lead to NFATc1 activation. Relative to non-irradiated cells, irradiated MSCs showed higher expression of NFATc1 at both mRNA and protein levels (Fig. 5F, G). NFATc1 is typically confined to the cytoplasm in a phosphorylated form. Upon stimulation, NFATc1 is dephosphorylated by calcineurin (CaN), a Ca^2+^-dependent phosphatase, and translocate into the nucleus to regulate the gene expression [21, 22].

In this study, we observed increased NFATc1 activation in the irradiated MSCs, indicated by enhanced translocation of NFATc1 from the cytoplasm to the nucleus (Fig. 5H), compared to non-irradiated cells. Increased cytoplasm expression of Fis1 was also detected (Fig. 5H), implicating the role of NFATc1 in regulating the Fis1 expression. Furthermore, we tested if NFATc1 stimulated mitochondrial fission by regulating the expression of Fis1. Using siRNA to inhibit *Nfatc1* expression (*p* < 0.001) (Fig. S9A, B), we observed a marked reduction in both NFATc1 expression and activation, as indicated by decreased nuclear translocation and cytoplasmic levels compared to the control and IR groups. Notably, NFATc1 inhibition in irradiated MSCs partially reversed radiation-induced NFATc1 activation, nuclear translocation, and Fis1 expression (*p* < 0.05) (Fig. 5H and Fig. S9C-E). We further examined the effects of NFATc1 inhibition on mitochondrial morphology and MSC functions. As expected, inhibiting NFATc1 alleviated radiation-induced mitochondrial fission (*p* < 0.05) (Fig. S10A, B) and rescued mitochondrial function in irradiated MSCs, as evidenced by reduced mitochondrial fission, decreased oxidative stress, improved oxidative phosphorylation, and increased ATP production (*p* < 0.05) (Fig. S10C-G).

Next, we performed ChIP assay to determine if NFATc1 directly binds to the *Fis1* promoter to regulate the transcription of this gene. The JASPAR database predicted that NFATc1 might be positively bound to the *Fis1* gene promoter at the -1868 bp to -1877 bp site with the specific sequence of GAAAGGAAAA (Fig. 5I). ChIP assays were conducted using the anti-NFATc1 or IgG antibodies to determine the binding sites on the *Fis1* promoter. A relatively higher signal was observed with the anti-NFATc1 antibody than with the IgG antibody (*p* < 0.001) (Fig. 5J, K), indicating that NFATc1 could bind to the *Fis1* gene promoter at the specific sequence to activate *Fis1* transcription. To fully determine that NFATc1 acts as the upstream of Fis1, we further constructed an overexpression vector of NFATc1 and transfected it into MSCs. The results showed that overexpression of NFATc1 could significantly upregulate the expression of Fis1 and promote mitochondrial fission in irradiated MSCs (*p* < 0.05) (Fig. S11).

Collectively, these findings have provided insights into the molecular mechanisms underpinning mitochondrial fragmentation following radiation. Notably, we showed that excessive mitochondrial fission induced by radiation, leading to mitochondrial fragmentation was mediated by increased Fis1 expression through activation of the Ca^2+^-CaN-NFATc1 pathway.

### Inhibition of Fis1 partially rescue radiation-induced bone injury

**Fig. 6:**
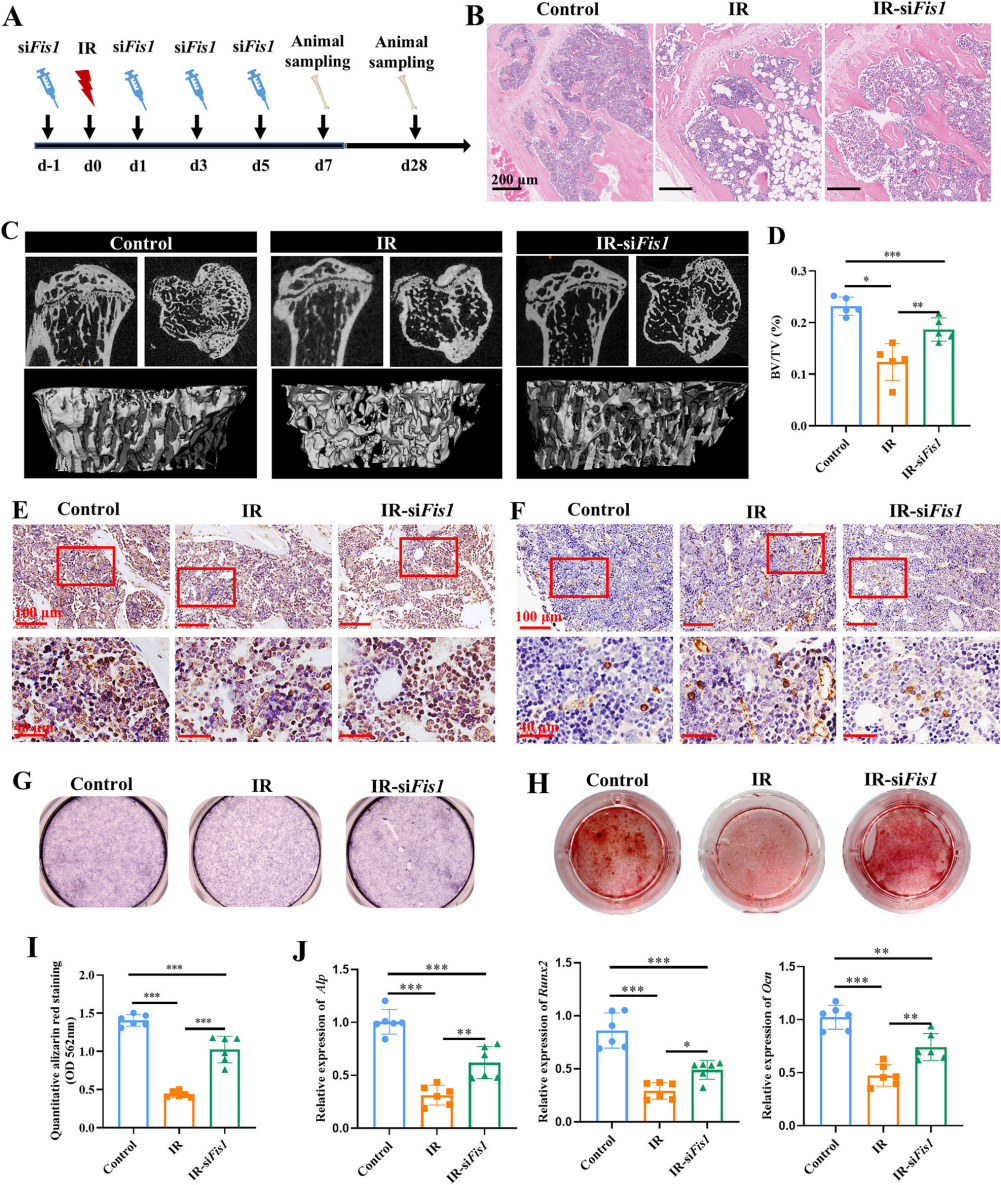
In vivo administration of *Fis1-siRNA* improved bone loss and osteogenic function of MSCs in irradiated mice. **A** Schematic showing intramedullary administration of *Fis1*-siRNA in a mouse model of radiation-induced bone injury. **B** H&E staining of the tibia. Scale bars, 200 µm. **C** Representative micro-CT images of the tibia showing the sagittal and top views (top panel) and the ROI (bottom panel). **D** Quantitative analysis of BV/TV. Immunohistochemical staining and semi-quantitative analysis of OCN (**E**) and PPAR-γ stained areas (**F**). Representative images. Scale bars, 200 or 40 µm. Bone marrow-derived MSCs were harvested from tibia of control and IR mice and evaluated for their osteogenic differentiation capacity, including ALP activity measurement (**G**), ARS staining (**H**) and semi-quantification of eluted dye (**I**) and qPCR analysis of mRNA levels of *Alp, Runx2, Ocn* (**J**). Mean ± SD, *p < 0.05, **p < 0.01, ***p < 0.001. Statistical analyses were determined by one-way ANOVA followed by Tukey’s multiple comparison test.

Next, we evaluated the in vivo effects of Fis1 inhibition on radiation-induced bone injury in a mouse model (Fig. 6A). The side effects of si-*Fis1* administration on major organs (heart, liver, spleen, lung, and kidney) in animal model were firstly studied, and histological staining revealed no obvious signs of toxicity associated with si-Fis1 treatment compared to control groups (Fig. S12A), demonstrating safety of si*-Fis1* application in vivo. In vivo imaging showed the localization of Cy5-labeled siRNA in the tibia, confirming the successful administration of siRNA for Fis1 inhibition (Fig. S12B). Subsequently, to verify Fis1 inhibition, tibial samples were harvested for immunohistochemical staining for Fis1 on day 7 following irradiation. IR-si*Fis1* mice displayed a significantly lower IOD for Fis1 staining in the bone marrow as compared with that of IR mice (*p* < 0.05) (Fig. S12C, D). Similarly, western blot analysis confirmed reduced Fis1 expression in siRNA-treated mice compared to IR-treated controls (Fig. S12E).

To further examine the downstream effects of Fis1 inhibition, tibia samples were collected on day 28 following irradiation. Relative to IR mice, IR-si*Fis1* mice showed more trabecular bone with lesser trabecular spacing and reduced adiposity, but still exhibit morphological differences when compared to that of control (non-irradiated) mice (Fig. 6B). This was confirmed by micro-CT evaluation that recorded significant improvements in bone parameters, including BV/TV (*p* < 0.05), Tb.N (*p* < 0.05) and BMD (*p* < 0.05) in IR-si*Fis1* mice, as compared with the IR mice (Fig. 6C, D and Fig. S12F). Despite these improvements, IR-si*Fis1* mice still exhibit significantly lower BV/TV and Tb.N than that of control mice (*p* < 0.05) (Fig. 6C, D and Fig. S12F). Immunohistochemical staining analysis further showed that IR-si*Fis1* mice had significantly higher IOD for OCN in the bone marrow, as compared with the IR mice (*p* < 0.05) (Fig. 6E and Fig. S12G). In contrast, a significantly lower IOD for PPAR-γ was measured in the IR-si*Fis1* mice, as compared with the IR mice (*p* < 0.01) (Fig. 6F and Fig. S12H). These findings demonstrate that Fis1 inhibition remarkably enhanced bone formation and reduced bone marrow adiposity, that were otherwise adversely impacted by radiation.

**Fig. 7:**
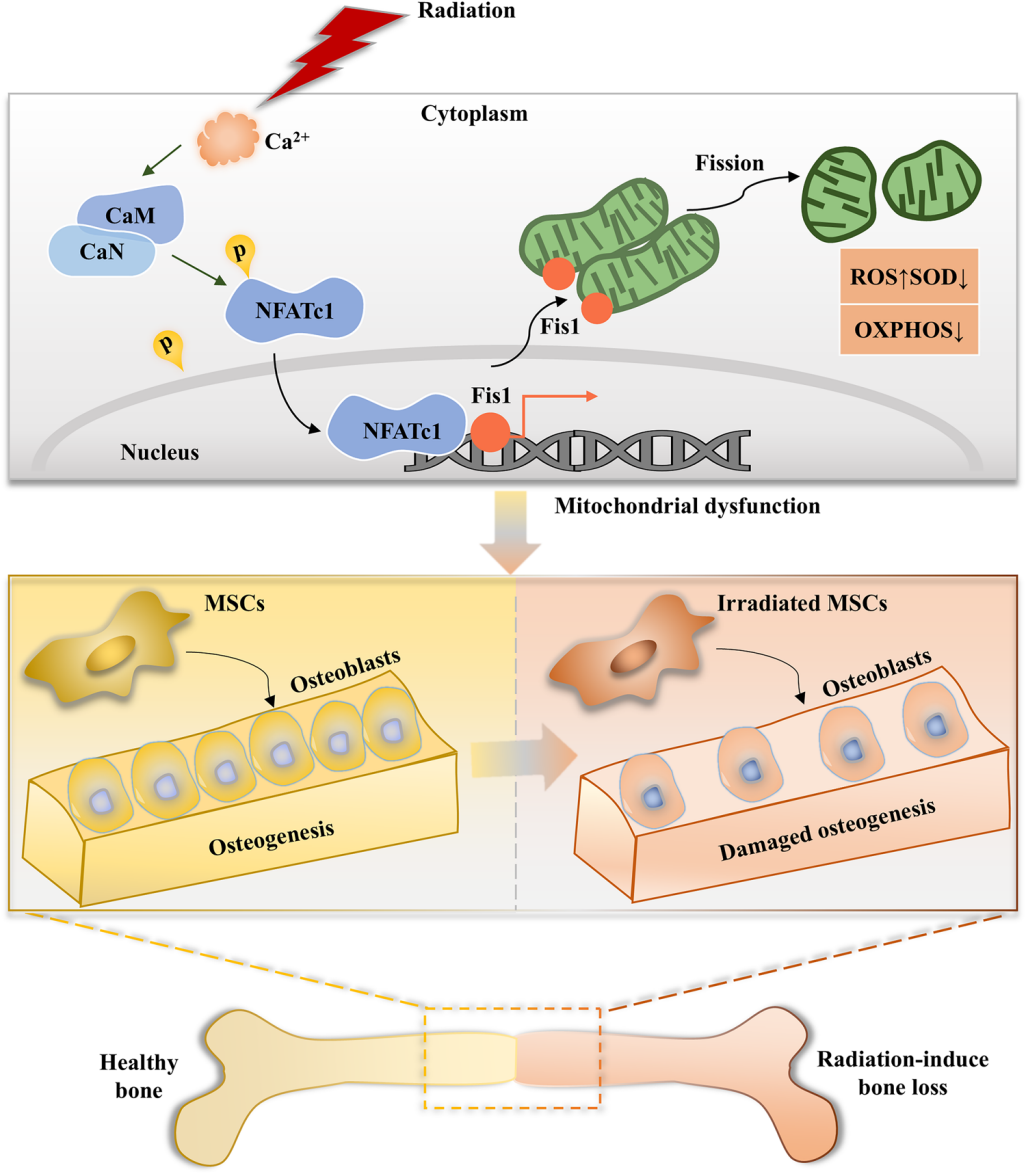
Radiation activates Ca^2+^/CaN/NFATc1/Fis1 signal to regulate mitochondrial homeostasis and osteogenesis. Radiation elevates cytosolic Ca²^+^, activating calcineurin (CaN). CaN dephosphorylates NFATc1, promoting its nuclear translocation where it upregulates Fis1 transcription, triggering excessive mitochondrial fission. Excessive mitochondrial fission mediated by Fis1 impairs mitochondrial function, as evidenced by reduced antioxidant capacity, suppressed oxidative phosphorylation, and diminished ATP production. These changes collectively inhibit MSC osteogenesis and ultimately contribute to bone loss.

Next, MSCs were isolated from the tibia of mice on Day 7 following radiation, and cultured in osteogenic medium. Consistent with our above findings that observed enhanced bone formation with *siFis1* treatment, compared with MSCs from IR mice, MSCs from IR*-siFis1* mice showed enhanced osteogenic differentiation, as evidenced by more intense staining for ALP and calcium (Fig. 6G, H) and a higher level of calcium deposition (*p* < 0.001) as compared to that from IR mice (Fig. 6I). Significantly higher mRNA levels of *Alp (p* < 0.01*) Runx2 (p* < 0.05*) and Ocn* (*p* < 0.01) were also detected in MSCs derived from IR*-siFis1* mice, as compared to those derived from IR mice (Fig. 6J). Overall, our findings demonstrate that radiation activates Fis1 via the Ca²⁺-CaN-NFATc1 pathway, triggering excessive mitochondrial fission, which impairs OXPHOS, ATP synthesis, and antioxidant capacity, collectively compromising MSC osteogenesis and bone homeostasis (Fig. 7). Inhibition of Fis1 inhibition can partially reverse these effects of radiation, and enhance MSC osteogenesis and bone repair.

## Discussion

Mitochondrial dysfunction has been increasingly implicated in impaired osteogenesis, the process of bone formation, following radiotherapy [23]. However, the molecular mechanisms underlying mitochondrial dysfunction that impairs osteogenesis remain to be fully clarified. Our study, for the first time, reveals the critical role of Fis1 in regulating the mitochondrial dynamics and MSC osteogenesis in radiation-induced bone injury. Upon radiation, elevated Fis1 expression induces excessive mitochondrial fission, along with reduced capacities for OXPHOS, ATP synthesis, and antioxidant defense, that collectively impedes MSC osteogenesis in radiation-induced bone injury. This process involves increased calcium (Ca^2+^) influx that stimulates CaN to promote NFATc1 dephosphorylation and nuclear translocation, which in turn, activates the transcriptional expression of Fis1. Further investigation reveals that inhibition of Fis1 remarkably alleviates the detrimental effects of radiation on MSC osteogenesis in vitro and bone loss in vivo. Our study reveals a novel molecular mechanism underlying impaired osteogenesis in radiation-induced bone injury, highlighting the therapeutic potential of targeting Fis1 to alleviate radiation-induced bone loss.

Mitochondria are the organelles responsible for energy production and have an important role in regulating several cellular processes, including MSC osteogenesis [24]. During the process of osteogenic differentiation, MSCs switch metabolic pathways from glycolysis to OXPHOS, and this bioenergetic switch is particularly important for osteogenic differentiation of MSCs, as OXPHOS not only provides ATP but also stimulates β-catenin signaling critical for osteogenic differentiation [25, 26]. Increased OXPHOS level has also been observed during osteogenesis in vivo, indicating an increased mitochondrial biogenesis in differentiated MSCs [27]. These findings demonstrated that radiation disrupts mitochondrial homeostasis by suppressing OXPHOS, decreasing SOD activity, and elevating ROS production, leading to mitochondrial dysfunction and impaired MSC osteogenesis.

Mitochondrial dynamics, which involves the processes of fission, fusion, biogenesis and mitophagy, plays a critical role in maintaining mitochondrial homeostasis and function [18]. This prompted us to determine if mitochondrial dynamics was altered by radiation. We found that radiation induced excessive mitochondrial fission leading to fragmentation into numerous smaller mitochondria, and increased expression of mRNA associated with mitochondrial fission, including Drp1 and Fis1. However, at protein level, Fis1 but not p-Drp1(Ser616) was significantly upregulated following radiation. Although we observed a slight increase in p-Drp1(Ser616), statistical significance was not reached. This could arise because signaling intensity of transcriptional upregulation fails to consistently surpass the activation threshold required for Drp1 phosphorylated modification. Moreover, beyond the Ser616 phosphorylation site (a key regulator promoting mitochondrial fission [28]), additional phosphorylation sites [29] might counterbalance its effects, warranting further investigation in future studies.

Being a mitochondrial outer membrane protein, Fis1 plays a pivotal role in mitochondrial dynamics by regulating mitochondrial fusion and fission [30]. Fis1 is commonly described as a Drp1 adaptor that participates in mitochondrial fission [31]. In addition, Fis1 is found to inhibit GTPase activity of pro-fusion proteins, including Mfn1, Mfn2, and Opa1, thus promoting mitochondrial fragmentation [30]. Fis1 is reported to regulate glucose metabolic homeostasis in high-fat diet-fed mice, in which Fis1 activation promotes the accumulation of fumarate, a TCA cycle intermediate [32]. Based on our findings, radiation induced excessive mitochondrial fission mediated by Fis1, causing mitochondrial fragmentation, which then disrupted the OXPHOS efficiency and impaired the ATP production. Fis1 has been reported as a tether between mitochondria and endoplasmic reticulum (ER) by binding to the ER protein Bap31, thereby promoting fragmentation at ER-associated fission sites [33]. Fis1 also participates in downstream processes such as mitophagy, where it attenuates LC3 lipidation by interacting with the Rab7 GAP TBC1D15 [34]. Moreover, the Fis1-TBC1D15 complex on lysosomes can activate Drp1 to drive mitochondrial fission [35]. Further investigation also uncovered an oligomeric Mid51/Fis1 complex that mechanistically couples Drp1 and Rab7 GTP hydrolysis machinery at mitochondria-lysosome contact sites [36], underscoring the complex interplay between mitochondria and lysosome networks in cellular homeostasis and disease. However, whether radiation-induced, Fis1-mediated fission in MSCs involves cross-talk between different organelles and the underlying mechanisms remain to be investigated.

During the bone remodeling, Ca^2+^ has been identified as a vital second messenger that controls various cellular functions including proliferation, apoptosis, differentiation, gene transcription and metabolism [37, 38]. Previous study has demonstrated that cytosolic Ca²⁺ overload activates Miro1, a mitochondrial motility protein, thereby increasing donut-shaped mitochondria formation [39]. Critically, Ca²⁺-induced mitochondrial shape transition persists in Drp1 and Fis1 knockout cells [39], indicating Ca^2+^ signaling acts upstream of Drp1/Fis1. The activity of NFATc1 is regulated by Ca²⁺/calmodulin-dependent calcineurin (CaN) phosphatase [21, 22]. Research has demonstrated that Ca²⁺/NFATc1 signaling plays an important role in regulating DRP1 mediated mitochondrial fission and mitochondrial apoptosis [40].

In this study, we demonstrated for the first time that Fis1 mediated mitochondrial fission was regulated by the Ca^2+^-CaN-NFATc1 pathway. During the process, Ca²⁺ influx binds calmodulin, activating CaN. Activated CaN dephosphorylates NFATc1, triggering its nuclear translocation and subsequent Fis1 transcription. In addition, activation of Ca^2+^-NFATc1-Fis1 pathway induced mitochondrial dysfunction and osteogenic suppression of irradiated MSCs. Crucially, inhibition of either Fis1 or NFATc1 effectively rescued radiation-induced mitochondrial fission and mitochondrial dysfunction, indicating the vital role of the Ca^2+^-NFATc1-Fis1 pathway in maintaining the mitochondrial homeostasis in irradiated MSCs. Functionally, inhibition of Fis1 improved osteogenesis of irradiated MSCs and remarkably alleviated radiation-induced bone loss. Together, these results emphasized the pivotal role of Fis1 in radiation-induced mitochondrial fission and impaired osteogenesis.

Given the pivotal role of mitochondrial dynamics in many diseases, developing effective strategies to regulate mitochondrial fusion/fission may thus improve the disease outcomes. For example, allosteric activation of transglutaminase 2 regulates osteoblast differentiation by promoting mitochondrial elongation and increasing ATP production, which ameliorates osteoporosis in ovariectomized mice [41]. In another study, DRP1 knockdown or DRP1 inhibitor (Mdivi-1) suppresses osteoclast differentiation and alleviates ovariectomy-induced bone loss in vivo [42]. As MSC osteogenesis has been shown to involve predominantly mitochondrial fusion [43], inhibiting Fis1 to reduce excessive mitochondrial fission caused by radiation could therefore be an alternative strategy to restore the mitochondrial dynamics and enhance osteogenesis in radiation-induced bone injury, as evidenced by our study.

In this study, we have focused mainly on uncovering the role of Fis1 in radiation-induced mitochondrial dysfunction and impaired osteogenesis. Mitochondrial function is regulated by the well-coordinated balance of fusion and fission dynamics. Further studies are required to determine the effects of Fis1 inhibition on mitochondrial elongation and fusion, and how these could contribute to the overall restoration of mitochondrial dynamics, and the balance of osteogenesis and osteoclastogenesis in bone formation and remodeling. A detailed understanding of the role of Fis1 on mitochondrial dynamics, from both mechanistic and structural standpoints, is therefore required to develop effective Fis1-targeting strategies for various bone injuries and diseases.

Mitochondria critically orchestrate radiation-induced cell death beyond metabolic, redox and calcium regulation [9, 44]. Mitochondria act as central hubs for diverse death pathways, orchestrating not only classical apoptosis [45], but also newly characterized inflammatory modalities—including pyroptosis, ferroptosis, and autophagy-lysosome-dependent death [46, 47]. Notably, apoptosis frequently associates with excessive mitochondrial fission in MSCs [48]. Crucially, Fis1-mediated mitochondrial fission emerges as an important regulator for apoptosis. In glioma models, enhanced radiation sensitivity correlates with fission amplification where Fis1 inhibition alleviates mitochondrial apoptosis [40]. Furthermore, lipotoxicity-induced Fis1 upregulation drives mitochondrial fragmentation that promotes NLRP3-dependent pyroptosis [49]. Fis1 lactylation triggers excessive fission, inducing ATP depletion, ROS overproduction, and mitochondrial catastrophe [50]. These findings strongly implicate that Fis1-mediated fission could act as a potential master switch governing apoptosis in irradiated MSCs. Although radiation-induced alterations in MSC differentiation potential have been widely confirmed, apoptotic responses to radiation—particularly the regulatory mechanisms mediated by mitochondrial dynamics—remain largely unexplored. While multiple studies report suppressed MSC proliferation at doses >4 Gy [16, 51, 52], others indicate preserved stemness even at 10 Gy [53], indicating complex death susceptibility. Further investigation is warranted to define the relationship between radiation-induced apoptosis and Fis1-mediated mitochondrial fission in MSCs.

In conclusion, our study reveals the critical role of Fis1 in regulating mitochondrial dynamics and MSC osteogenesis in radiation-induced bone injury. We show that radiation activates Fis1 expression via the Ca^2+^-CaN-NFATc1 pathway, which induces excessive mitochondrial fission, along with reduced capacities for OXPHOS, ATP synthesis, and antioxidant defense, that collectively impedes MSC osteogenesis and bone repair in radiation-induced bone injury. Importantly, Fis1 inhibition alleviates these detrimental effects of radiation on MSC osteogenesis and bone loss, highlighting the therapeutic potential of targeting Fis1 in radiation-induced bone loss.

## Supplementary information

Supplementary Material

Full and uncropped western blots

## Data Availability

The data used and analyzed in this study are available upon reasonable request from the corresponding author.
