# Supplementary material for: Mitochondrial dysfunction in mesenchymal stem cells impairs osteogenesis in radiation-induced bone injury via Ca2+-NFATc1-Fis1 pathway
Source: Cell Death Dis. 2025 Dec 2;17(1):69. doi: 10.1038/s41419-025-08281-w (PMC12828000; doi:10.1038/s41419-025-08281-w)
Supplement: Supplementary file 1 — Supplementary Material [file 41419_2025_8281_MOESM1_ESM.docx]

**Supplementary Material**

**Mitochondrial dysfunction in mesenchymal stem cells impairs osteogenesis in radiation-induced bone injury via Ca^2+^-NFATc1-Fis1 pathway**

Lin Ren ^a, b, 1^, Xiaodan Chen ^a, b, 1^, Ying Zheng ^a, b, 1^, Jiayan Li ^a, b^, Jiali Yu ^a, b^, Linlin Ou ^a, b^, Gen Liu ^a, b^, Bin Cheng ^a, b,^ *, Wei Seong Toh ^c, d,^ *, Juan Xia ^a, b,^ *

^a^ Hospital of Stomatology, Guanghua School of Stomatology, Sun Yat-sen University, Guangzhou, 510055, China

^b^ Guangdong Provincial Key Laboratory of Stomatology, Sun Yat-sen University, Guangzhou, 510055, China

^c^ Department of Orthopaedic Surgery, Yong Loo Lin School of Medicine, National University of Singapore, 119228, Singapore

^d^ Singapore Faculty of Dentistry, National University of Singapore, 119085, Singapore

**This file includes:**

Supplementary Figures. 1-12

**Fig. S1.** Radiation impaired the trabecular bone structure.

**Fig. S2.** Expression of MSC surface marker CD44 in control and irradiated MSCs.

**Fig. S3.** Evaluation of osteogenic potential of MSCs under different radiation doses.

**Fig. S4.** Radiation induced mitochondrial fission and upregulated the expression of Fis1 *in vitro*.

**Fig. S5.** Radiation induced oxidative stress injury and disrupted mitochondrial respiration in MSCs *in vitro*.

**Fig. S6.** Knockdown of Fis1 significantly down-regulated the expression of Fis1 in MSCs *in vitro.*

**Fig. S7.** Inhibition of Fis1 rescued the osteogenesis of irradiated MSCs.

**Fig. S8.** RNA-seq assay suggested the activation of Ca^2+^/CaN/NFATc1 pathway in irradiated MSCs *in vitro*.

**Fig. S9.** Knockdown of NFATc1 alleviated the effects of Fis1 in irradiated MSCs *in vitro*.

**Fig. S10.** Inhibition of NFATc1 rescued mitochondrial fission and dysfunction of irradiated MSCs.

**Fig. S11.** NFATc1 overexpression upregulated the expression of Fis1 and increased mitochondrial fission in irradiated MSCs *in vitro*.

**Fig. S12.** The administration of si-*Fis1* alleviated radiation-induced bone loss *in vivo*.

Supplementary Table 1

**Table S1** Sequences of primers for qPCR


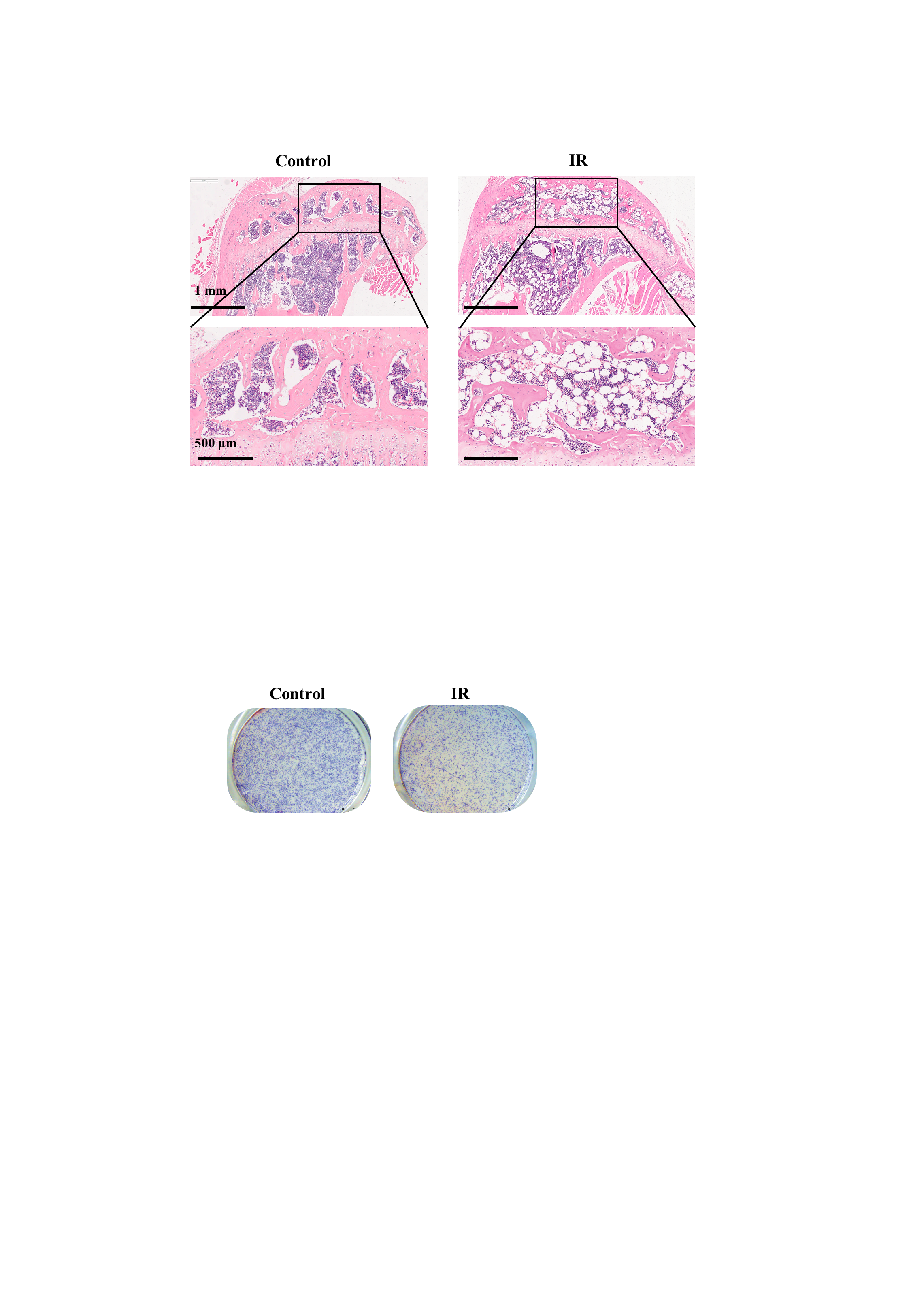


**Fig. S1.** Radiation impaired the trabecular bone structure. Representative images of H&E staining revealed thinning of bone struts within the trabecular bone, and increase in bone porosity and bone marrow adiposity in the IR group as compared to the control group. Scale bars, 1 mm or 500 µm.


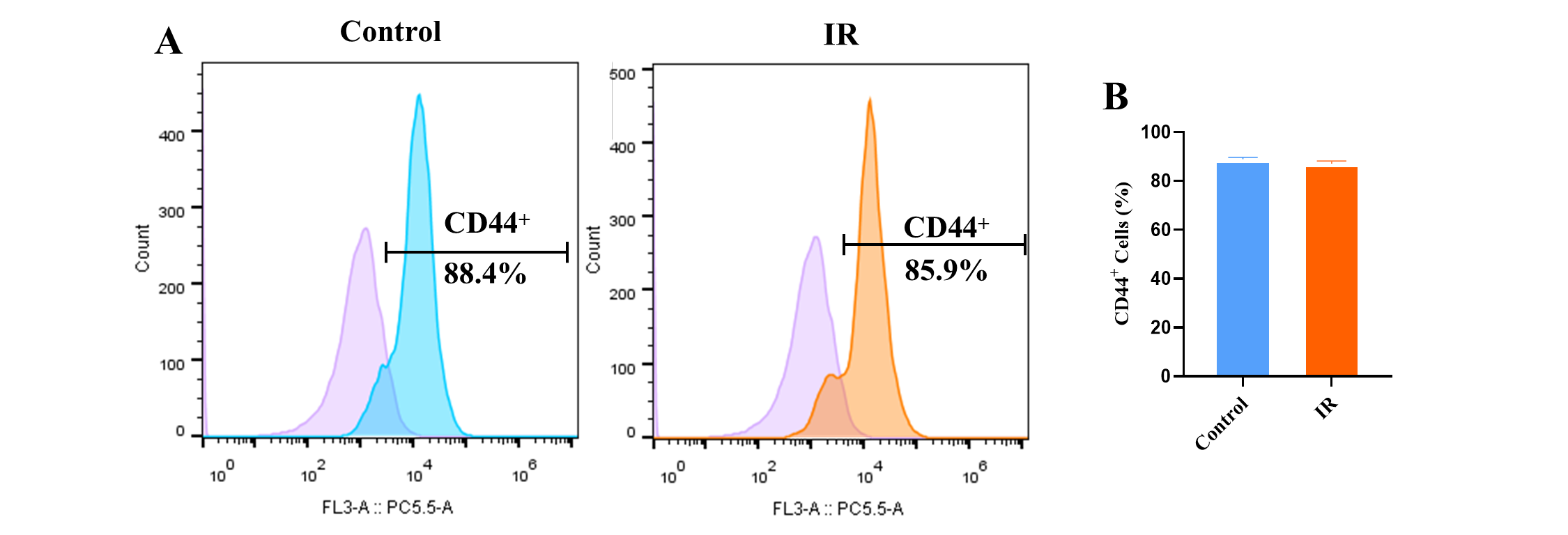


**Fig. S2.** Expression of MSC surface marker CD44 in control and irradiated MSCs. **A, B** Flow cytometry analysis **(A)** and quantification of CD44-positive cells (**B)**.


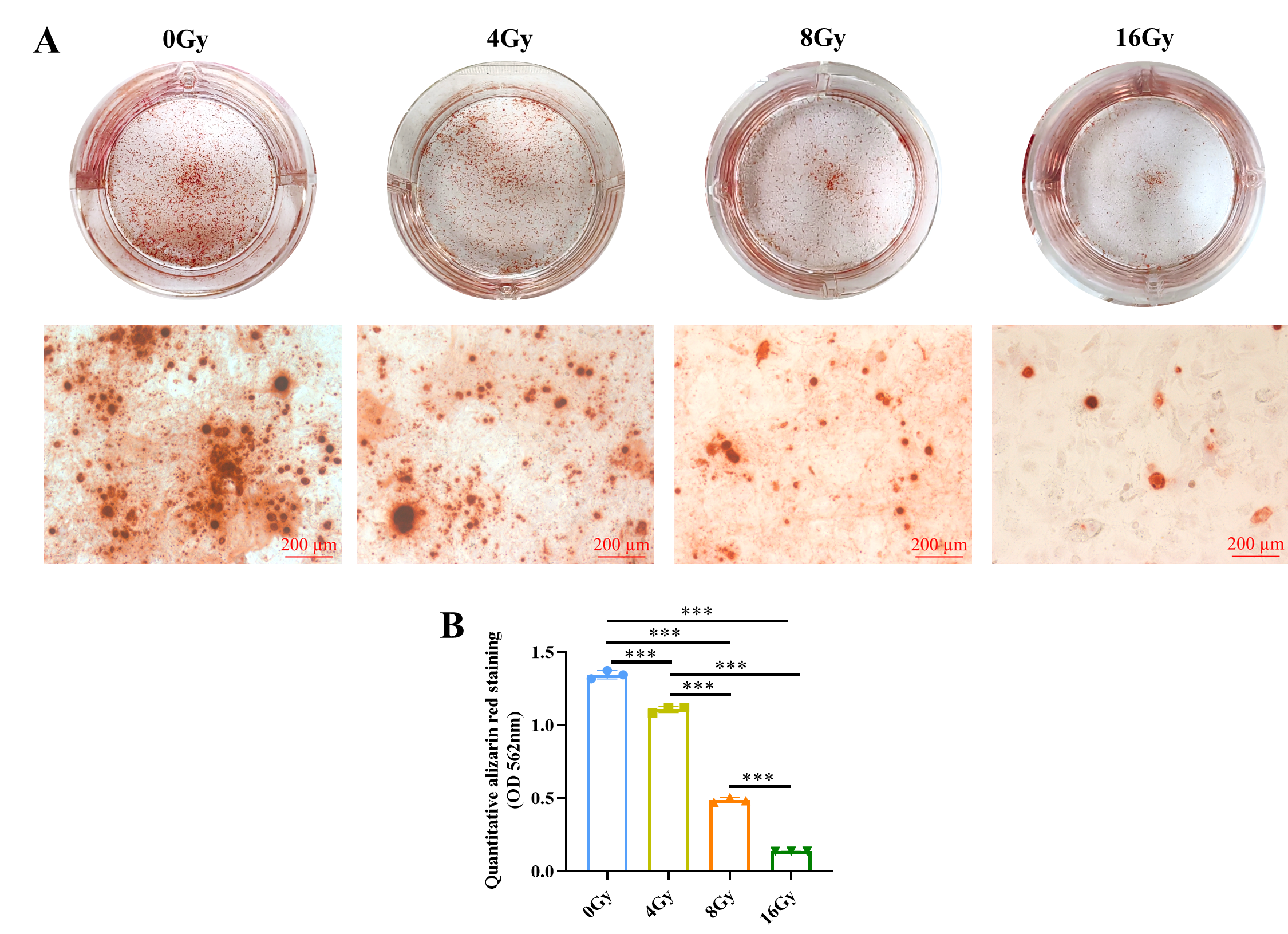


**Fig. S3.** Evaluation of osteogenic potential of MSCs under different radiation doses. **A** General and microscopic images of alizarin red staining (ARS). **B** Semi-quantification of ARS staining. Scale bars, 200 µm. Data are presented as mean ± SD, ****p* < 0.001. Statistical analyses were determined by one-way ANOVA followed by Tukey’s multiple comparison test.


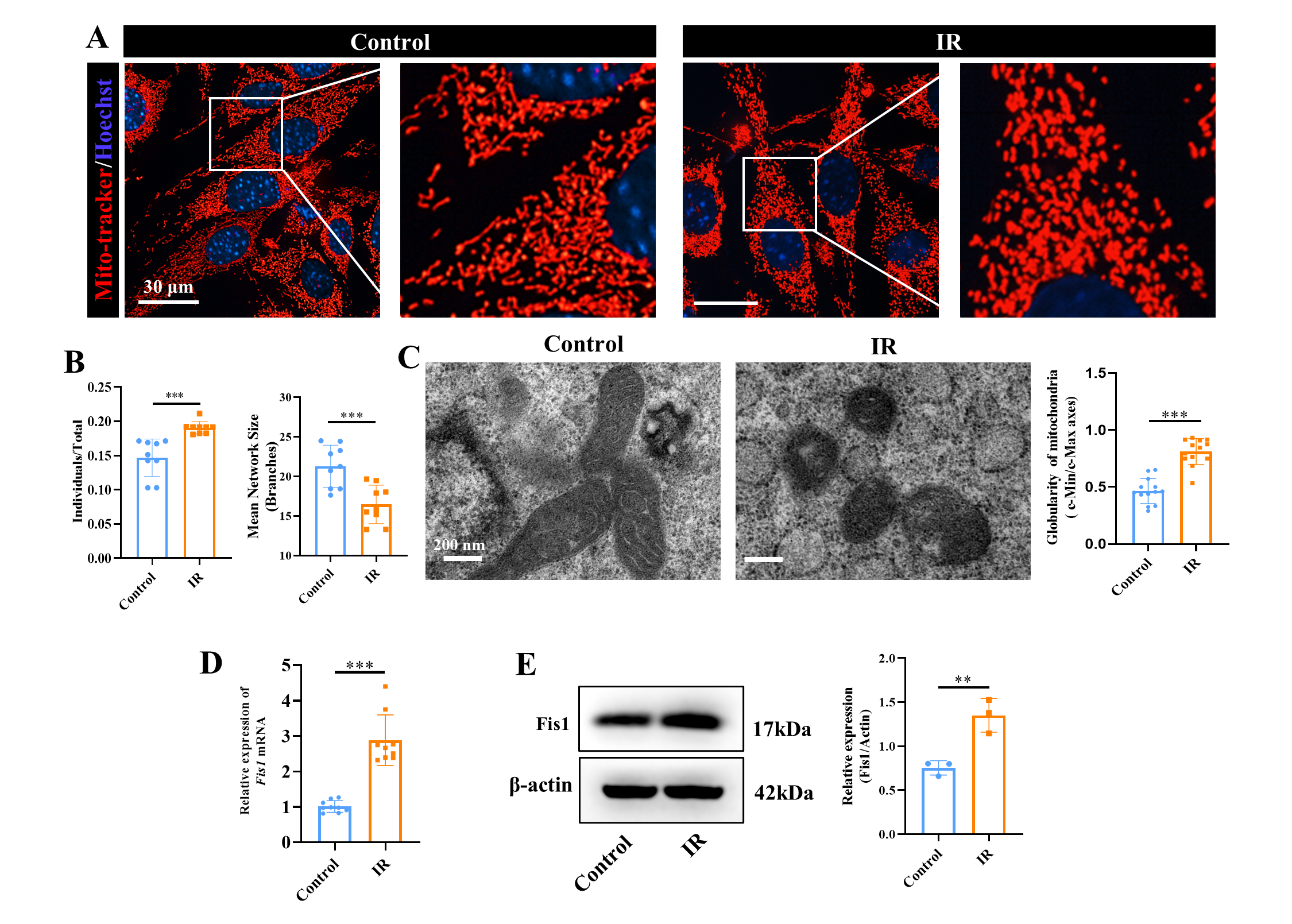


**Fig. S4.** Radiation induced mitochondrial fission and upregulated the expression of Fis1 *in vitro*. **A** MitoTracker™ Red staining and analysis of mitochondrial morphology. Scale bar, 30 µm. **B** Quantitative analysis of mitochondrial individuals (left) and mitochondrial network (right). **C** Representative TEM images of mitochondrial morphology and analysis of mitochondrial globularity. Scale bars, 200 nm. **D** qPCR analysis of *Fis1* mRNA expression. **E** Western blotting and semi-quantitative analysis of Fis1 protein expression. Data are presented as mean ± SD, ***p* < 0.01, ****p* < 0.001. Statistical analyses were determined by unpaired Student’s *t*-test.


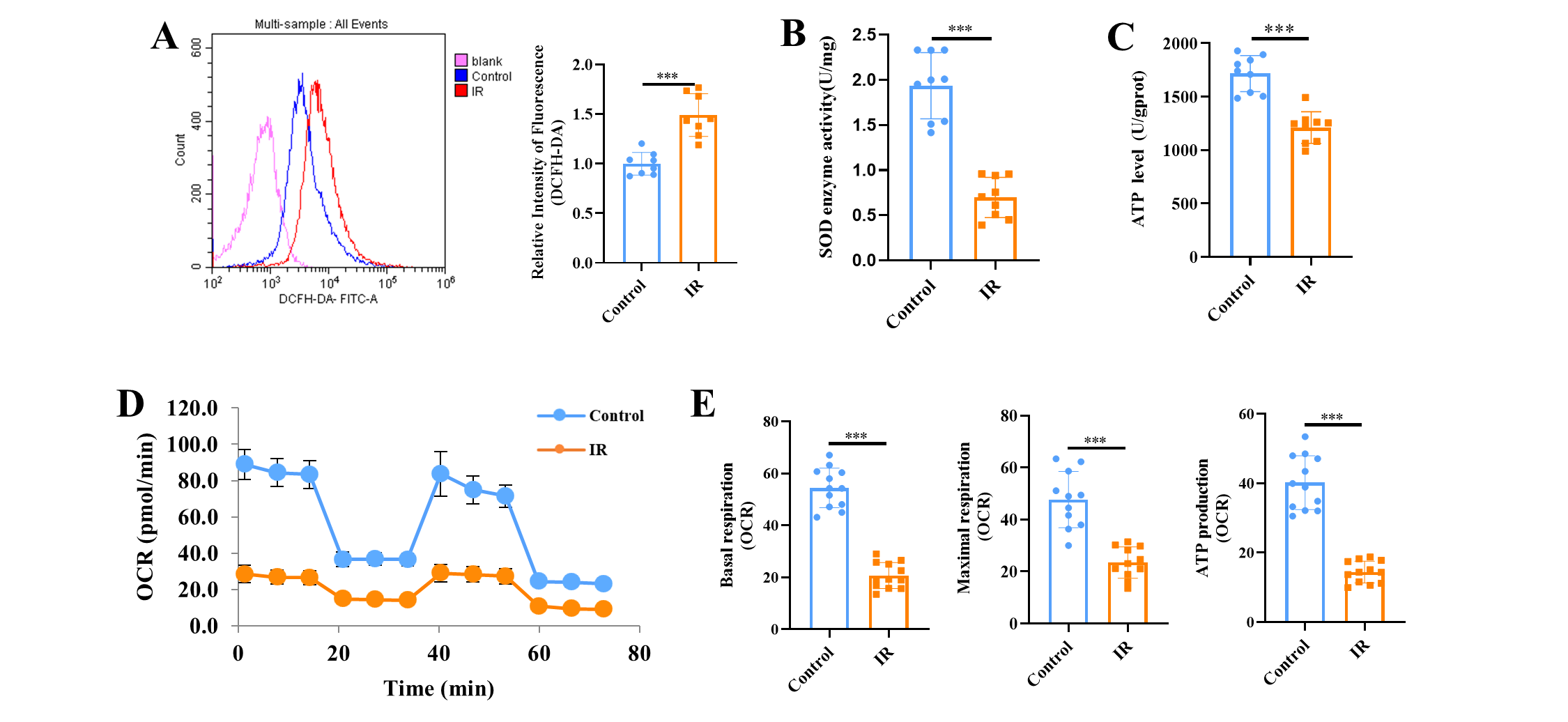


**Fig. S5.** Radiation induced oxidative stress injury and disrupted mitochondrial respiration in MSCs *in vitro*. **A** ROS level was evaluated using DCFH-DA fluorescent assay detected by flow cytometry. **B** Analysis of SOD enzyme activity. **C** Quantitative measurement of ATP level. **D, E** Metabolic analysis using Seahorse analyzer showing OCR curve and quantification of basal respiration, maximal respiration and ATP-linked respiration. Data are presented as mean ± SD, ****p* < 0.001. Statistical analyses were determined by unpaired Student’s *t*-test.


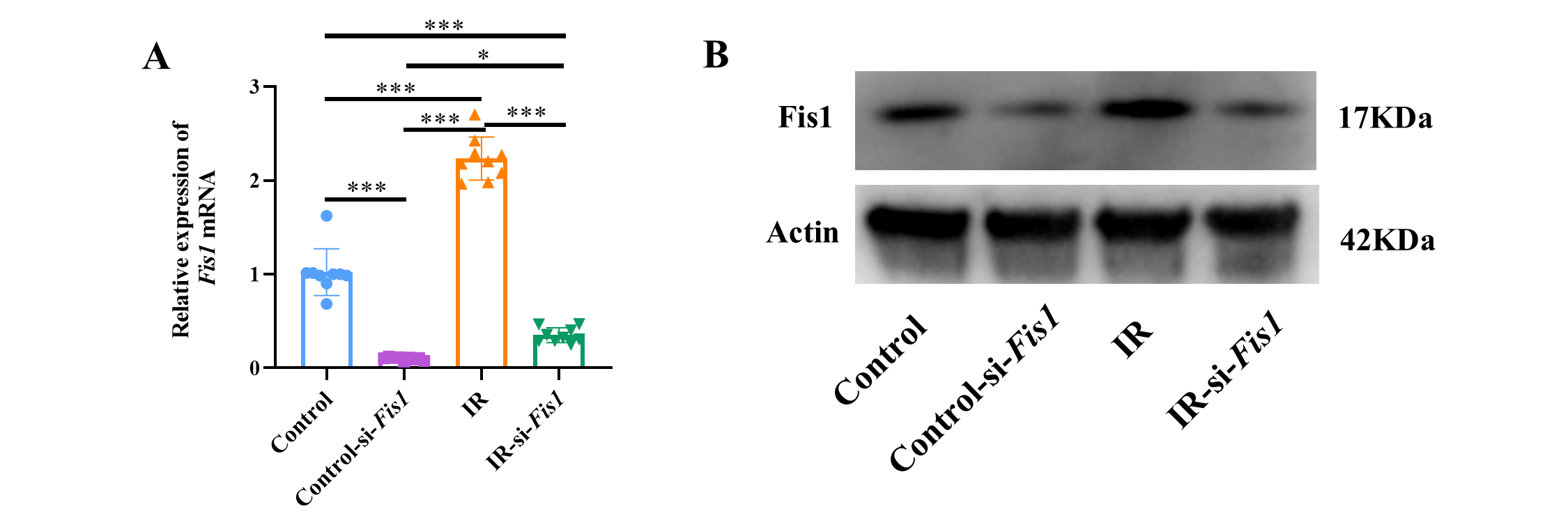


**Fig. S6.** Knockdown of Fis1 significantly down-regulated the expression of Fis1 in MSCs *in vitro.* **A B** Application of si-*Fis1* significantly down-regulated the mRNA **(A)** and protein **(B)** expression of Fis1 in MSCs. Data are presented as mean ± SD, **p* < 0.05, ****p* < 0.001. Statistical analyses were determined by one-way ANOVA followed by Tukey’s multiple comparison test.


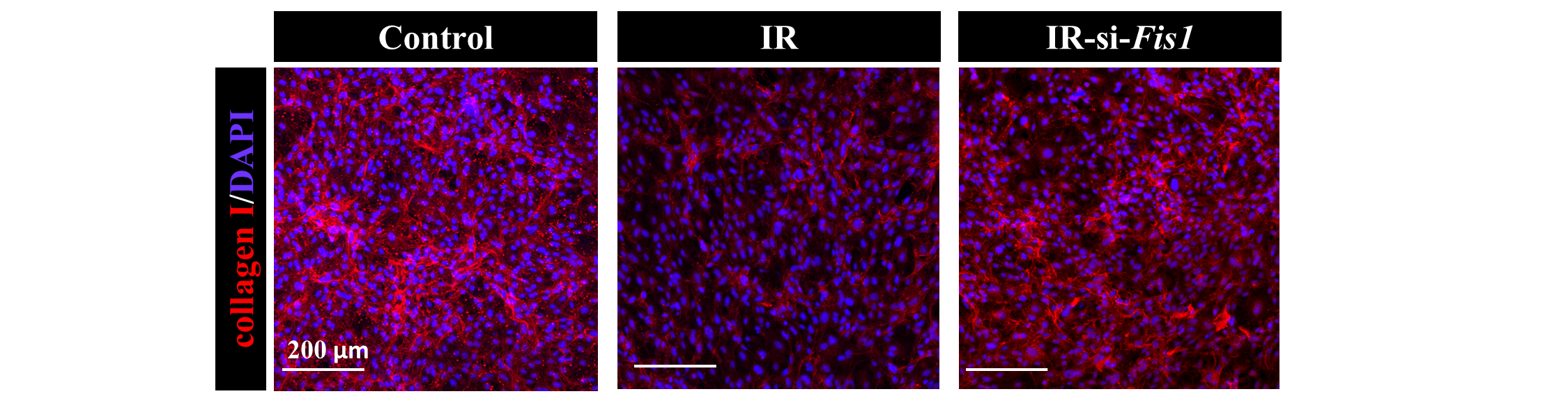


**Fig. S7.** Inhibition of Fis1 rescued the osteogenesis of irradiated MSCs. Irradiated MSCs treated with si-*Fis1* displayed enhanced expression of collagen I compared with IR cells. Scale bar, 200 µm.


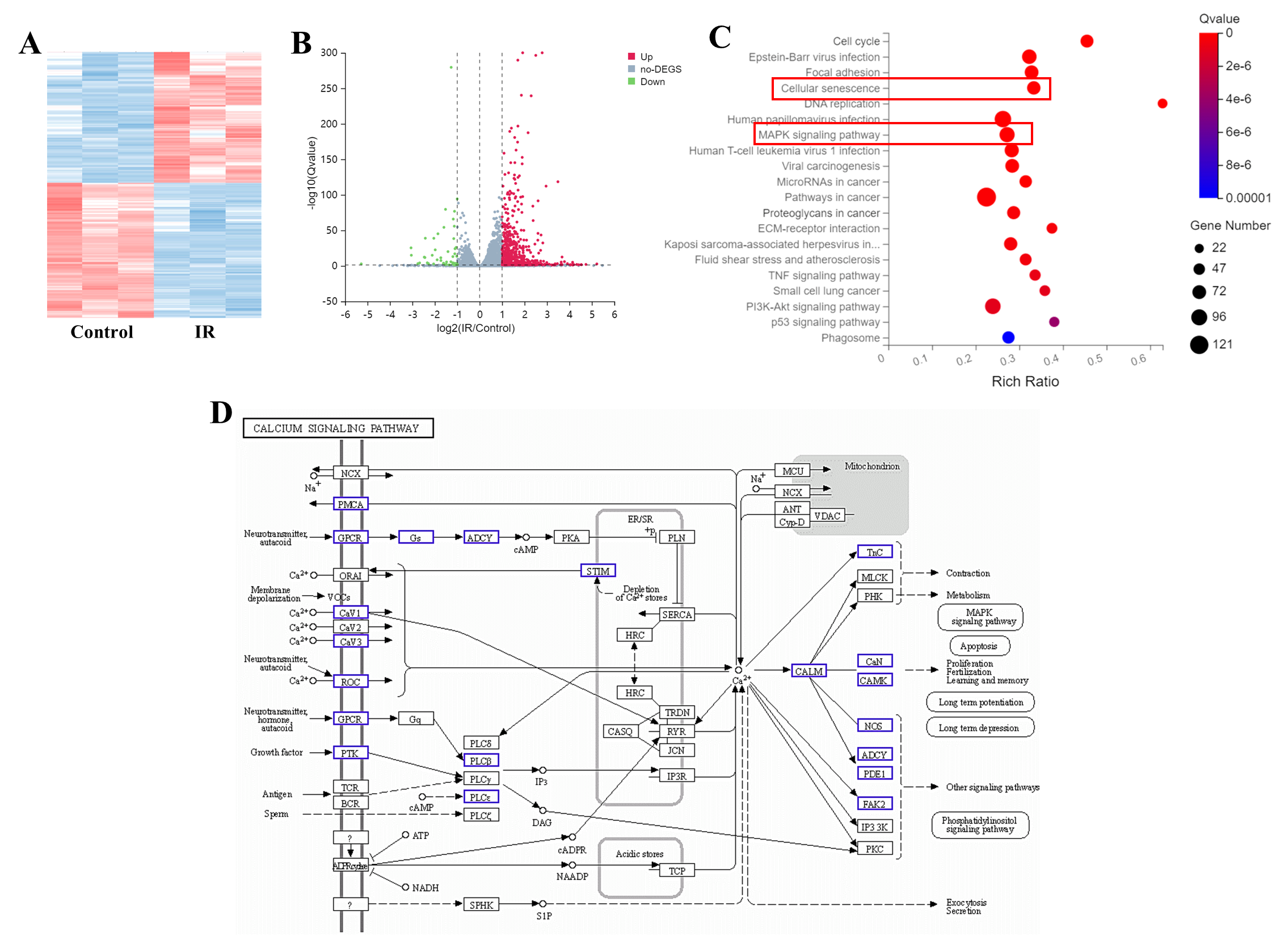


**Fig. S8.** RNA-seq assay suggested the activation of Ca^2+^/CaN/NFATc1 pathway in irradiated MSCs *in vitro*. **A** The clustering heat map of the whole gene of irradiated and non-irradiated MSCs. **B** Volcanic maps of differentially expressed genes. **C** KEGG enrichment of differential genes between irradiated and non-irradiated MSCs. **D** Changes in gene level of calcium signaling pathways based on the analysis of KEGG pathway map, the blue box showed the genes that were significantly upregulated in irradiated cells compared to non-irradiated cells.


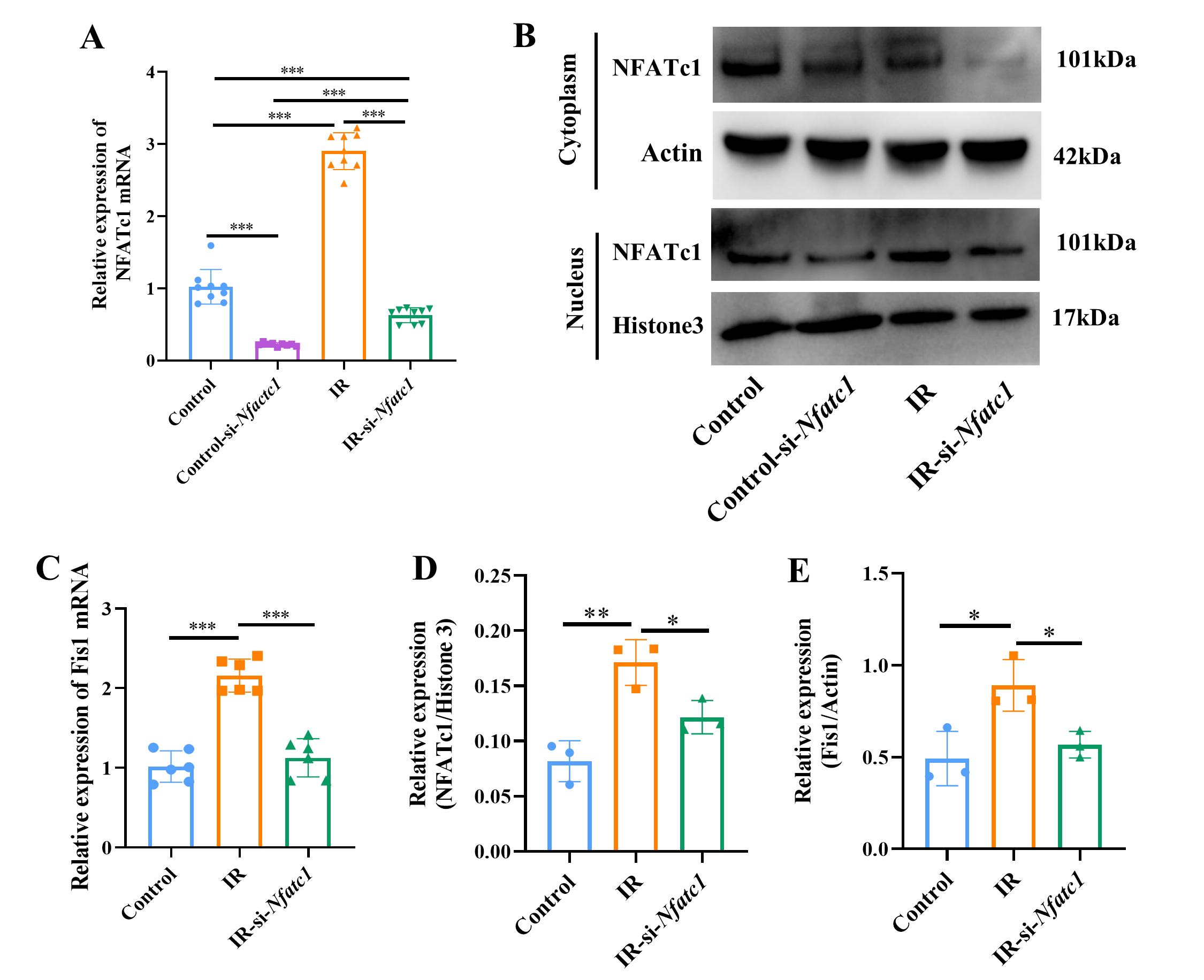


**Fig. S9.** Knockdown of NFATc1 alleviated the effects of Fis1 in irradiated MSCs *in vitro*. **A, B** Application of si-*Nfatc1* significantly down-regulated the mRNA **(A)** and protein **(B)** expression of NFATc1 in MSCs. **C-E** By means of siRNA inhibition of NFATc1, the expression of Fis1 and NFATc1 was analyzed, including qPCR analysis of *Fis1* level **(C)**. Quantitative analysis of protein expression of nuclear NFATc1 **(D)** and cytoplasmic Fis1 **(E)**. Data are presented as mean ± SD, **p* < 0.05, ***p* < 0.01, ****p* < 0.001. Statistical analyses were determined by one-way ANOVA followed by Tukey’s multiple comparison test.


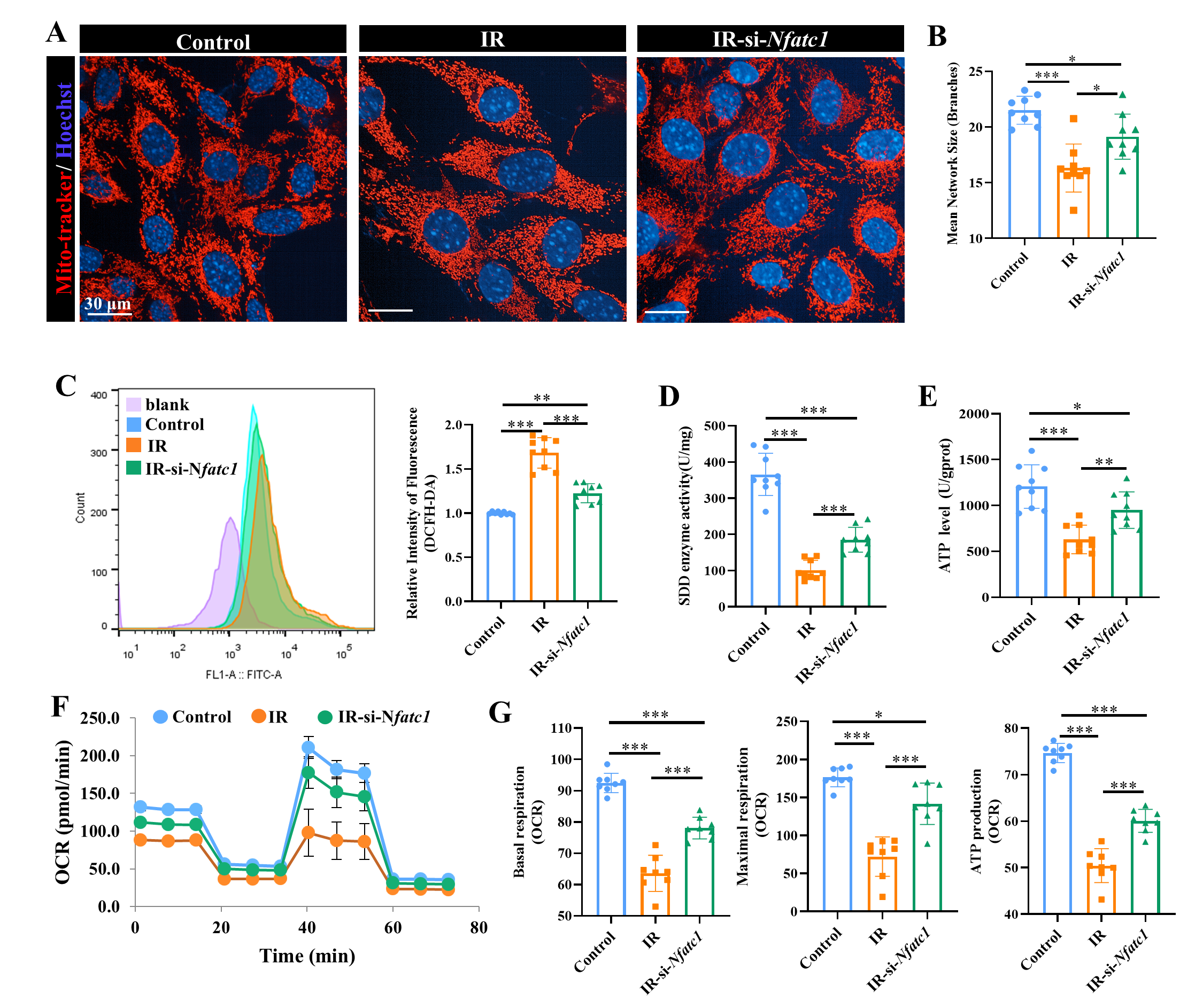


**Fig. S10.** Inhibition of NFATc1 rescued mitochondrial fission and dysfunction of irradiated MSCs. **A** MitoTracker™ Red staining of mitochondria in MSCs. Representative images. Scale bars, 30 µm. **B** Semi-quantitative analysis of mitochondrial network using ImageJ-MINA. **C** Analysis of ROS production by DCFH-DA fluorescent assay using flow cytometry. **D** Measurement of SOD activity. **E** Quantitative measurement of ATP level. **F, G** Metabolic analysis using Seahorse analyzer showing OCR curve (**F**) and quantification of basal respiration, maximal respiration and ATP-linked respiration (**G**). Mean ± SD, **p* < 0.05, ***p* < 0.01, ****p* < 0.001. Statistical analyses were determined by one-way ANOVA followed by Tukey’s multiple comparison test.


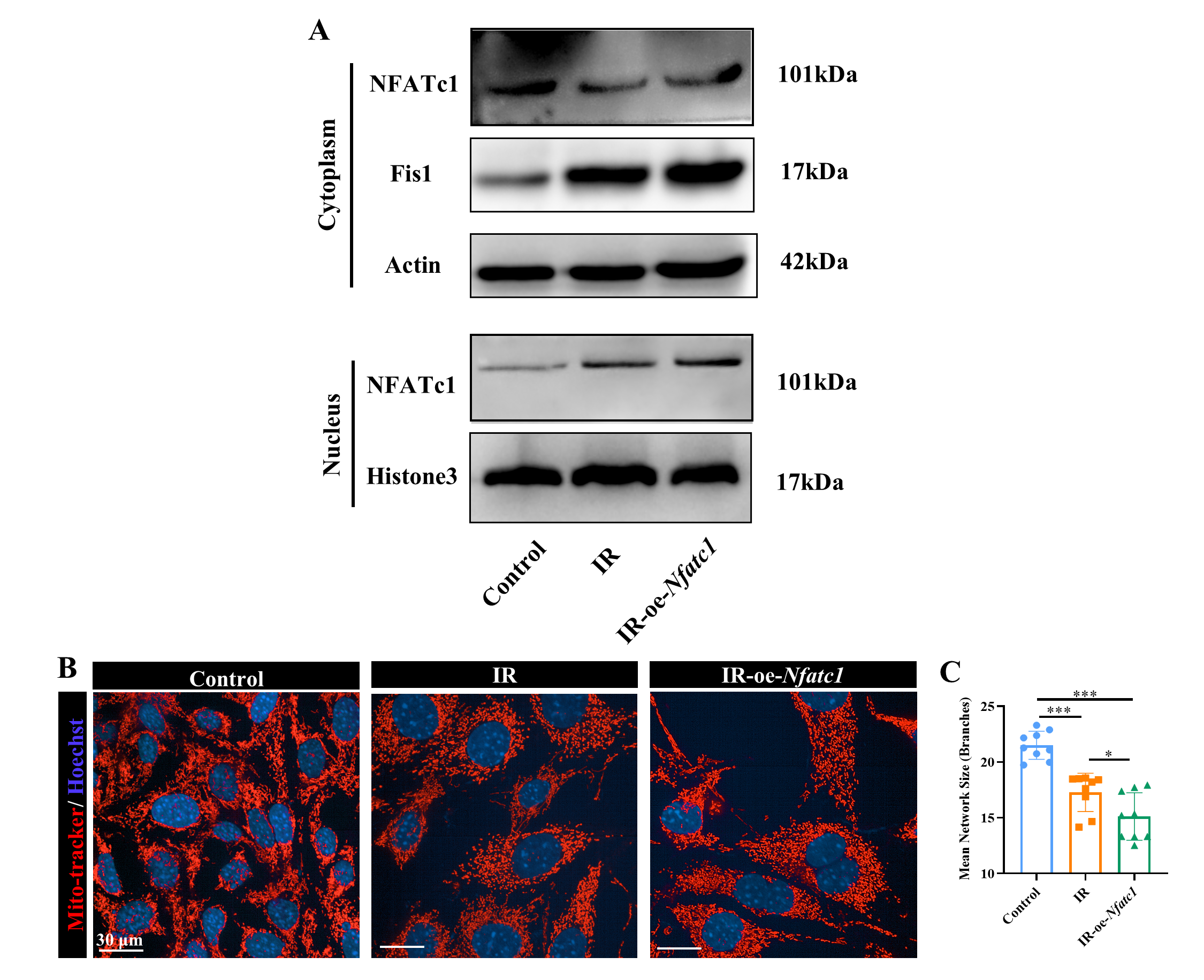


**Fig. S1****1.** NFATc1 overexpression upregulated the expression of Fis1 and increased mitochondrial fission in irradiated MSCs *in vitro*. **A** Western blot analysis of Fis1 and NFATc1 protein expression. **B** MitoTracker™ Red staining of mitochondria in MSCs. Representative images. Scale bars, 30 µm. **C** Semi-quantitative analysis of the mitochondrial network using ImageJ-MINA. Mean ± SD, **p* < 0.05, ****p* < 0.001. Statistical analyses were determined by one-way ANOVA followed by Tukey’s multiple comparison test.


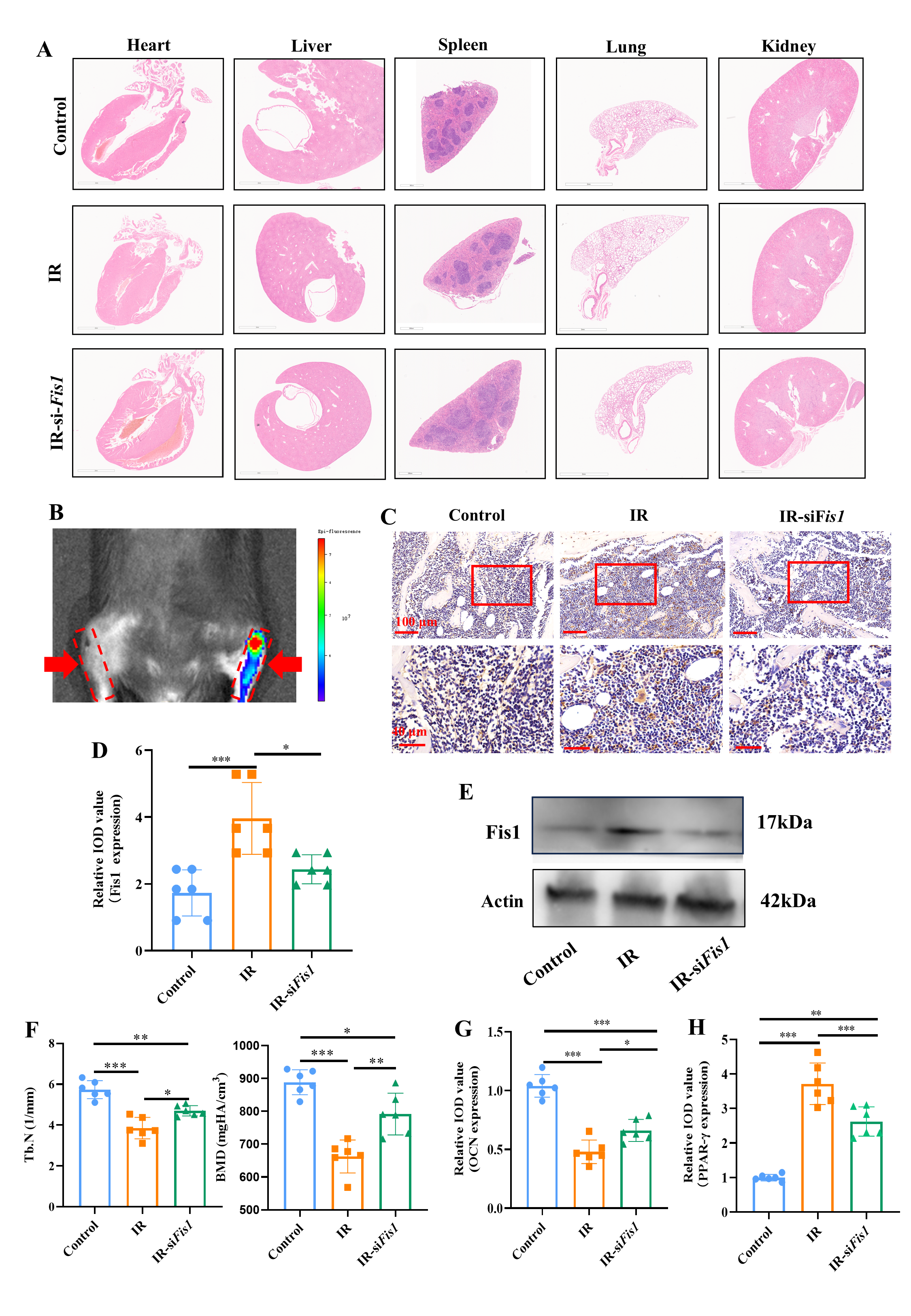


**Fig. S****12.** The administration of si-*Fis1* alleviated radiation-induced bone loss *in vivo*. **A** Hematoxylin and eosin (H&E) staining of tissue sections from the liver, spleen, lungs, and kidneys following si-*Fis1* administration. **B**. *In vivo* imaging demonstrated the distribution of Cy5-labeled siRNA. **C, D** Immunohistochemical staining for Fis1 within bone marrow. Scale bars, 100 or 40 µm. **E.** Western blot analysis of Fis1 protein expression. **F** Micro-CT analysis of trabecular bone number and bone mineral density. **G, H** Semi-quantitative analysis of the expression of OCN **(G)** and PPAR-γ **(H)** based on immunohistochemical staining. Data are presented as mean ± SD, **p* < 0.05, ***p* < 0.01, ****p* < 0.001. Statistical analyses were determined by one-way ANOVA followed by Tukey’s multiple comparison test.

The sense and antisense PCR primers used are listed in Table S1 as follows.

**Table S1 Sequences of primers for qPCR**

| Gene | Sequence of prime |
| --- | --- |
| *β-actin* | F：5’‑CTTTTCCAGCCTTCCTTCTTG‑3’ |
|  | R：5’‑TTGGCATAGAGGTCTTTACGG‑3’ |
| *Drp1* | F：5’‑CAGGAATTGTTACGGTTCCCTAA‑3’ |
|  | R：5’‑CCTGAATTAACTTGTCCCGTGA‑3’ |
| *Fis1* | F：5’‑TGTCCAAGAGCACGCAATTTG‑3’ |
|  | R：5’‑CCTCGCACATACTTTAGAGCCTT‑3’ |
| *Mff* | F：5’‑ATGCCAGTGTGATAATGCAAGT‑3’ |
|  | R：5’‑CTCGGCTCTCTTCGCTTTG‑3’ |
| *Alp* | F：5’‑CCCACACTCAAGGGAGAGGT ‑3’ |
|  | R：5’‑GGAGGATTCCAGATACAGGCA ‑3’ |
| *Runx2* | F：5’‑TTTAGGGCGCATTCCTCATC‑3’ |
|  | R：5’‑TGTCCTTGTGGATTAAAAGGACTTG‑3’ |
| *Ocn* | F：5’‑ATGGCAGAAACGGTATCTCCA‑3’ |
|  | R：5’‑CTCGGATGCTATTCGATCAAGTT‑3’ |
| *Nfatc1* | F：5’‑CCGTTGCTTCCAGAAAATAACA‑3’ |
|  | R：5’‑TGTGGGATGTGAACTCGGAA‑3’ |
| *Can* | F：5’‑CAGAGGGTGCTTCGATTCTC‑3’ |
|  | R：5’‑CCCCTAAGAAGAGGTAGCGA-3′ |
