# Supplementary material for: Mitochondrial dysfunction in mesenchymal stem cells impairs osteogenesis in radiation-induced bone injury via Ca2+-NFATc1-Fis1 pathway
Source: Cell Death Dis. 2025 Dec 2;17(1):69. doi: 10.1038/s41419-025-08281-w (PMC12828000; doi:10.1038/s41419-025-08281-w)

Fig. 3F

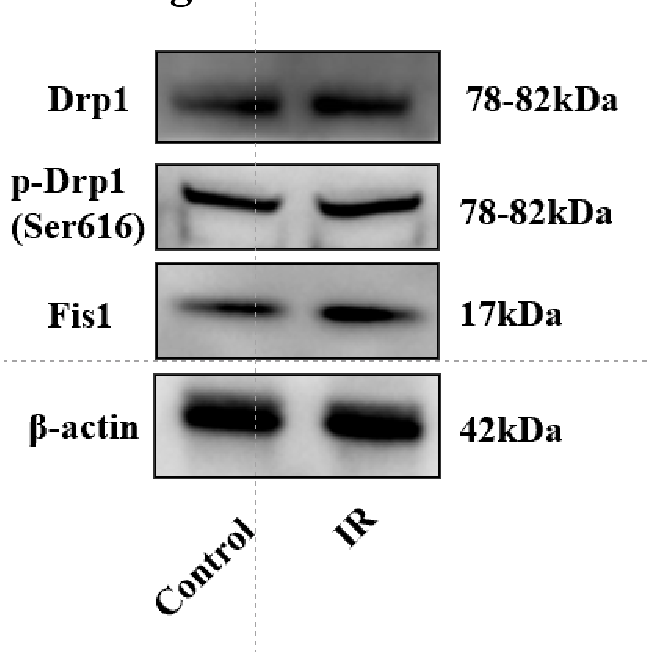

Full and uncropped western blots

Drp1

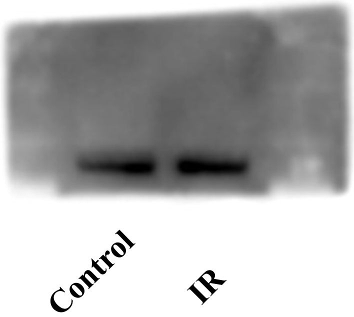

p-Drp1
(Ser616)

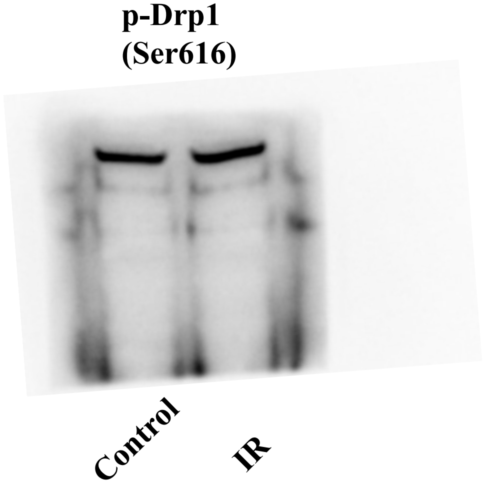

Fis1

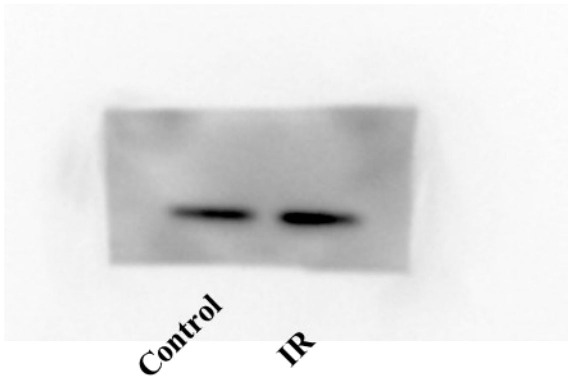

Actin

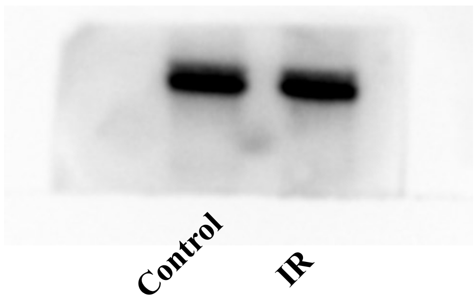

Fig. 5H

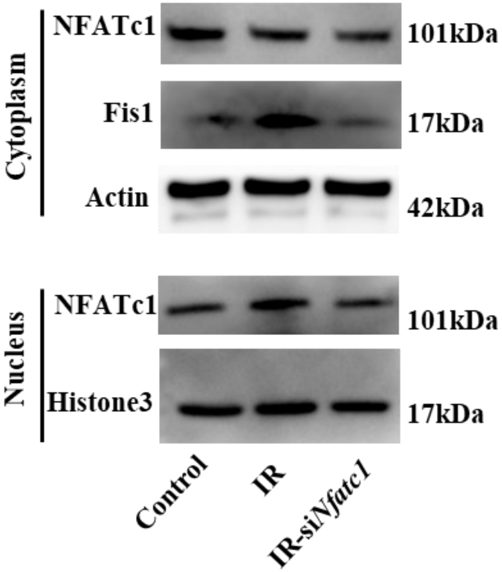

Full and uncropped western blots

NFATc1 (Nucleus)

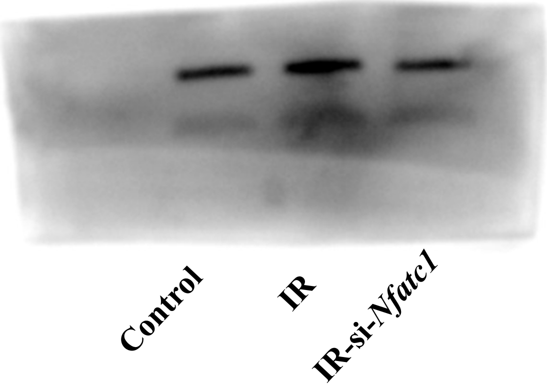

Histone3 (Nucleus)

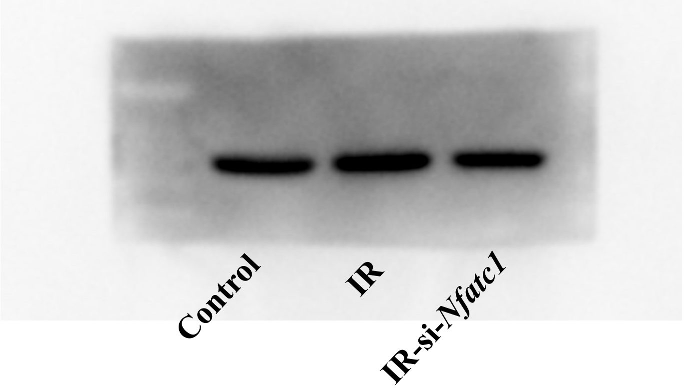

NFATc1 (Cytoplasm)

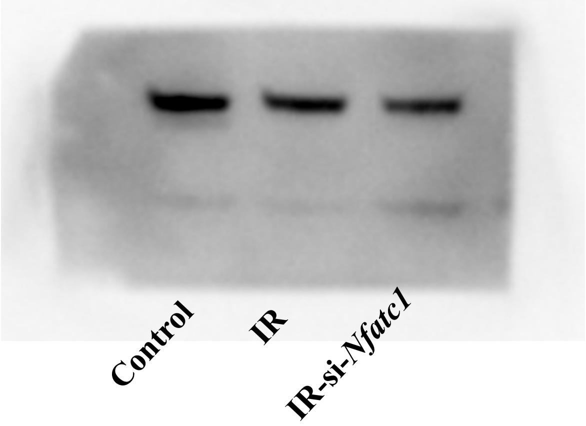

Fis1 (Cytoplasm)

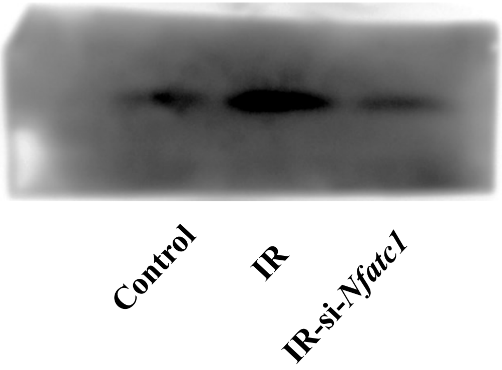

Actin (Cytoplasm)

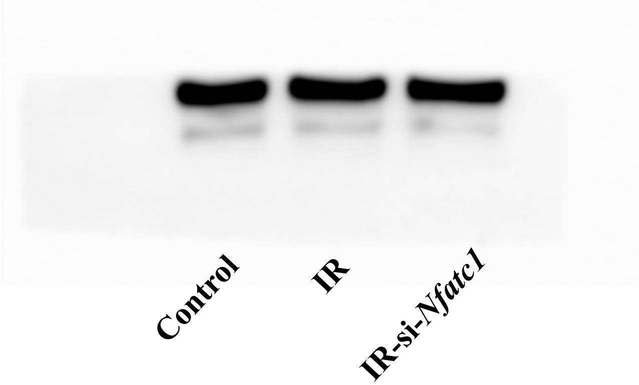

Fig. S4E

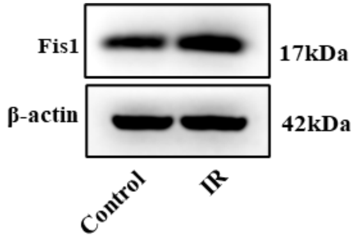

Full and uncropped western blots

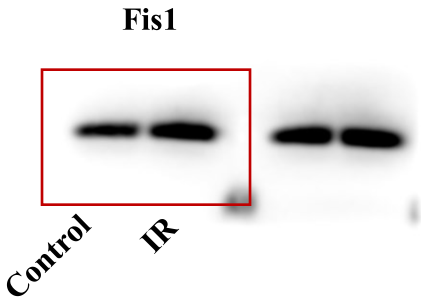

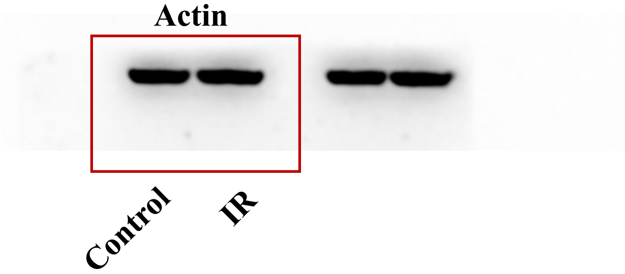

Fig. S6B

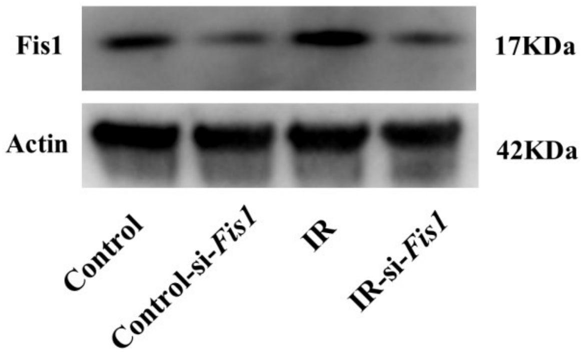

Full and uncropped western blots

Fis1

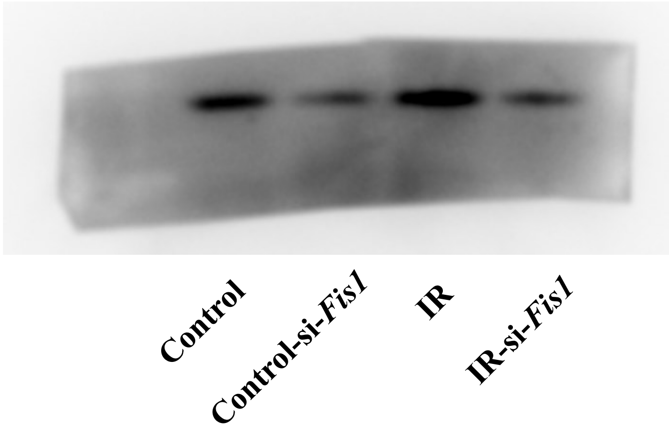

Actin

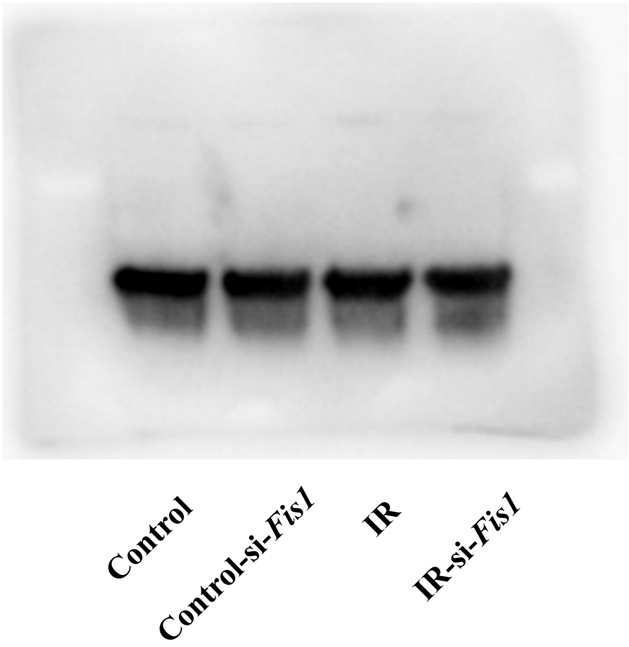

Fig. S9B

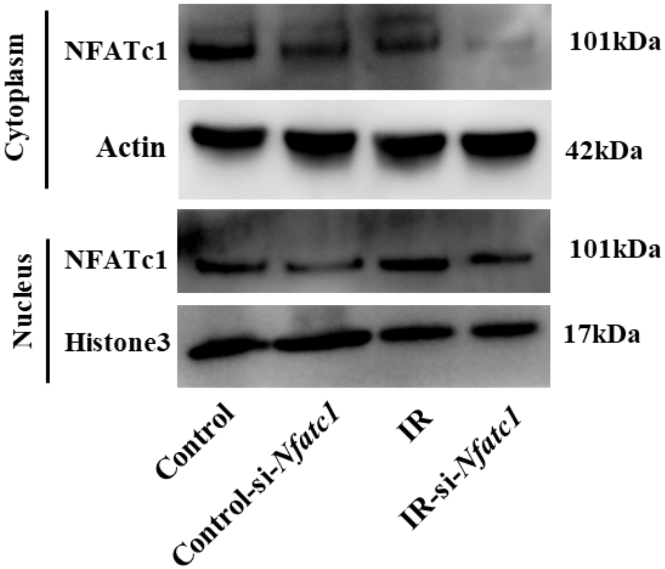

Full and uncropped western blots

NFATc1 (Cytoplasm)

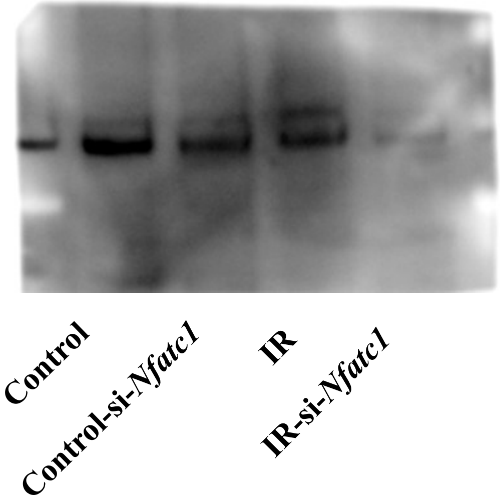

NFATc1 (Nucleus)

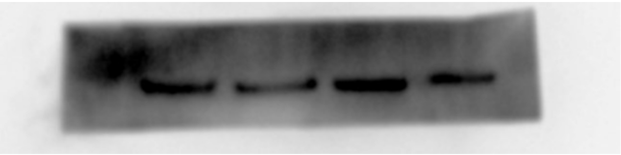

Histone3 (Nucleus)

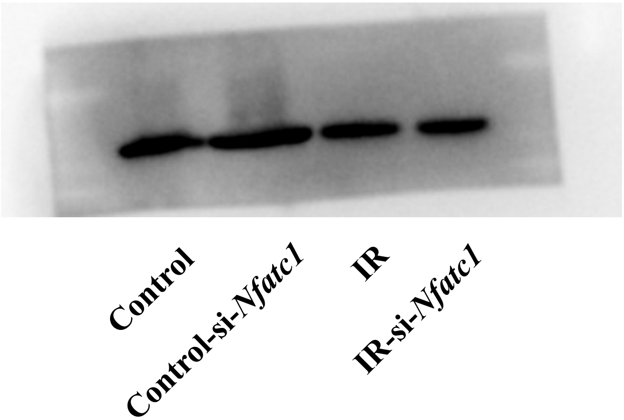

Actin (Cytoplasm)

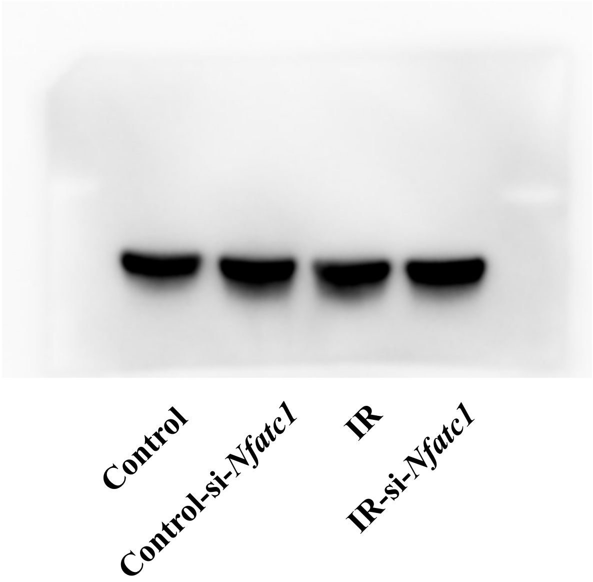

Fig. S11A

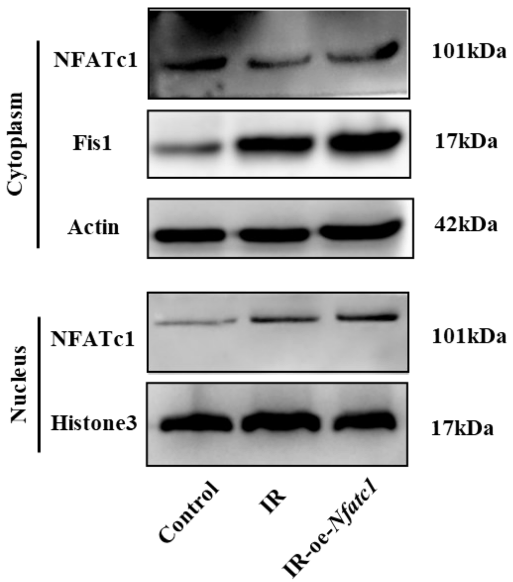

Full and uncropped western blots

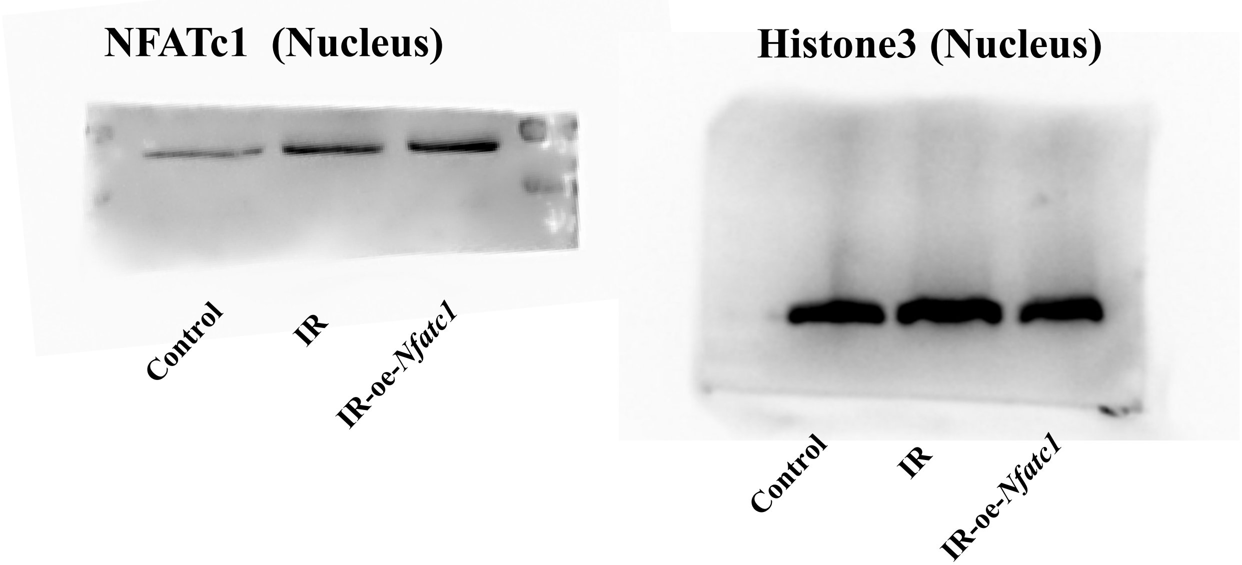

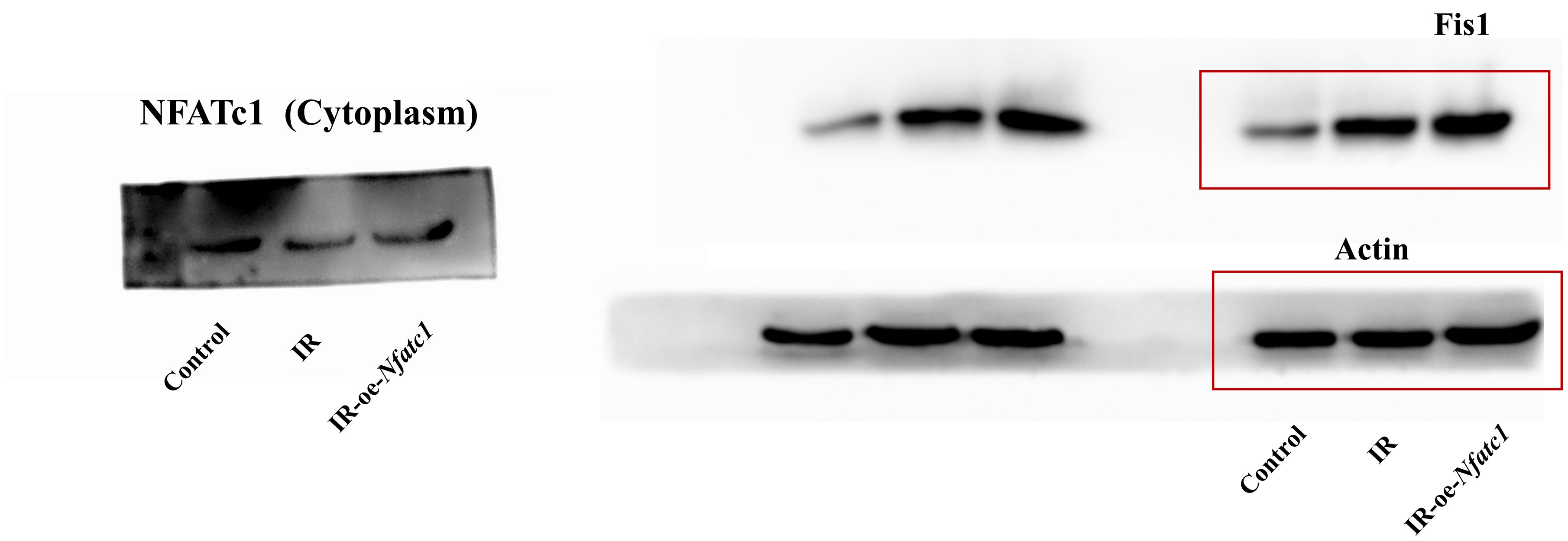

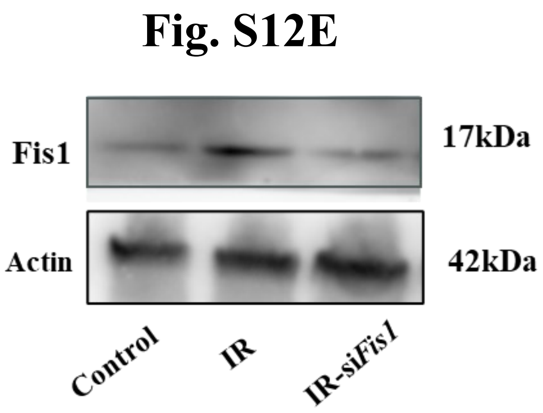

Full and uncropped western blots

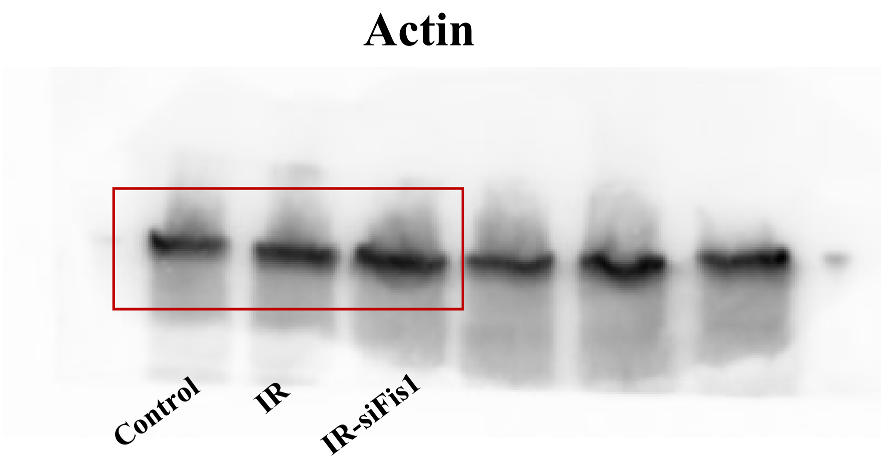

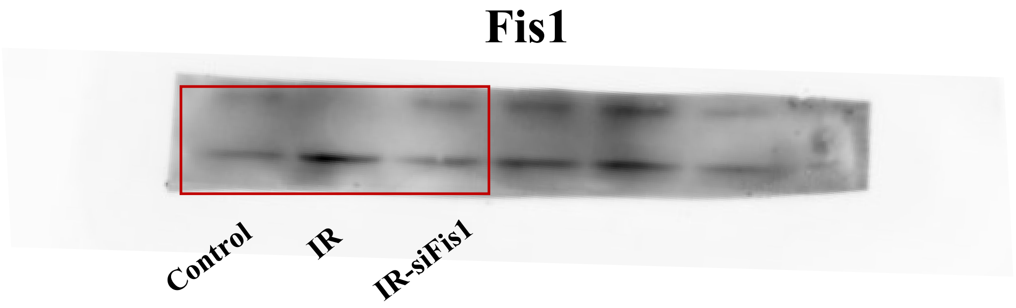

Supplement: Supplementary file 2 — Full and uncropped western blots [file 41419_2025_8281_MOESM2_ESM.pdf]
